# CT-PestNet: A novel crop pest classification model based on cross-scale feature distillation enhancement and vision transformer with local token interaction

**DOI:** 10.1371/journal.pone.0356061

**Published:** 2026-08-14

**Authors:** Youming Li, Weilin Cao, Liqiang Zhang

**Affiliations:** School of Artificial Intelligence, Neijiang Normal University, Neijiang, Sichuan, China; Shijiazhuang Tiedao University, CHINA

## Abstract

The continuous growth of the global population has led to an increasing demand for food, yet the annual food losses caused by pests are immeasurable. Therefore, accurately identifying crop pests and adopting effective control strategies is of great practical significance. In recent years, intelligent crop pest classification models based on deep learning have made significant progress, which can be primarily divided into two categories: convolutional neural network (CNN)-based and Transformer-based. However, the ability of CNN is limited in modeling the relationship between long-distance regions in images; the capability of Transformer is insufficient in capturing the local discriminative features of crop pests, which leads to unsatisfactory performance of the above two intelligent crop pest classification models. To address the above issues, this paper proposes a new crop pest classification model (CT-PestNet). First, CT-PestNet combines CNN and Transformer to fully leverage the former’s strengths in local detail extraction and the latter’s capabilities in modeling global dependencies. Second, a cross-scale feature distillation enhancement module (CFDE) is designed to respond to the challenges of diverse pest morphologies (with varying sizes and shapes) and complex backgrounds. The CFDE employs the “distillation-then-enhancement” strategy to enhance the model’s perception of small or diverse pests, thereby improving its classification accuracy and robustness in complex backgrounds. Finally, a local token interaction module (LTIR) is constructed to overcome the insufficient modeling of adjacent token features in the Transformer encoder. The LTIR can achieve deep discriminative feature mining by enhancing the feature interaction among adjacent tokens, thereby improving the model’s processing ability and recognition performance for complex images. On the IP102 and D0 datasets, CT-PestNet achieves accuracies of 77.32% and 99.73%, respectively; especially on the more challenging IP102 dataset, it outperforms the best CNN-based model by 3.62% and the best Transformer-based model by 1.32%, fully proving its effectiveness.

## Introduction

The global population is projected to reach approximately 10 billion by 2050. However, limited arable land resources may make food security one of the major challenges facing the world. According to World Health Organization statistics, the annual crop loss rate caused by crop pests is as high as 40% [1]. In this context, accurately identifying crop pests and formulating scientific and efficient control strategies is of great significance for ensuring food security and improving agricultural productivity [2].

Traditional crop pest classification methods primarily rely on manual visual inspection and empirical judgment. This approach is not only inefficient and highly subjective, but also requires a high level of professional knowledge from the identifier, making it prone to misidentification and missed identifications [3]. More critically, it has become inadequate to meet the urgent needs of modern agriculture for pest classification in terms of efficiency and accuracy. In recent years, with the continuous improvement of computer computing power and the refinement of artificial intelligence theory, intelligent crop pest classification models based on deep learning have gradually emerged and achieved remarkable results [4–6]. Overall, existing intelligent crop pest classification models are primarily categorized into two types: convolutional neural network (CNN)-based and Transformer-based.

Since the CNN can effectively extract local features and spatial structural information from images through multi-layer convolutional stacking operations, many researchers have adopted variants of the CNN [7–10] as the backbone of intelligent crop pest classification models. For example, Ahmad et al. [11] employed the CNN to extract image features and combined machine learning algorithms for feature analysis and classification, achieving automated identification of various pests. Liu et al. [12] proposed a pest classification model based on transfer learning and CNN. This model was pre-trained on the ImageNet dataset using AlexNet, VGG-16, and ResNet-50, and the learned parameters were transferred to the pest dataset for training and testing. Zhang et al. [13] noted that most existing pest classification models need considerable computational overhead. Therefore, they proposed a low-energy hybrid ResNet architecture (AM-ResNet). Experimental results demonstrated that AM-ResNet reduced energy consumption by more than 40% while causing less than a 2% decrease in accuracy. Zheng et al. [14] developed a novel pest classification approach (PCNet) based on EfficientNet-V2. By incorporating a coordinate attention mechanism and a feature fusion module, PCNet effectively integrates shallow and deep features while mitigating information loss introduced by downsampling. Ayan et al. [15] presented a genetic algorithm-based hyperparameter optimization strategy to refine pre-trained CNNs for pest classification. The optimized model achieved accuracy rates of 99.89% and 71.84% on the D0 and IP102 datasets, respectively. Nandhini et al. [16] introduced a Visual Regenerative Fusion Network (VRFNet) to recover semantic details from distorted images, thereby enhancing its classification performance for crop pests. Although CNN-based intelligent crop pest classification models have achieved specific results, the CNN is limited in modeling the relationship between long-distance regions in images. This means that CNN-based models may fail to effectively capture the overall information of the image, thereby affecting their classification ability and reliability.

Compared to the CNN, the Transformer can better capture the long-range dependence relationship between different regions of images through its self-attention mechanism. Therefore, other researchers have chosen variants of the Transformer [17–20] as the backbone of intelligent crop pest classification models. For example, Qi et al. [21] combined the residual network with the Transformer to propose Detection Transformer (DETR). In DETR, a multihead criss-cross attention module (MCCA) was designed to capture the interrelationships among object queries, thereby enhancing the model’s adaptability to variations in pest morphology and size. Wang et al. [22] proposed a Transformer-based pest detection framework, RP-DETR. In this framework, the RepPConv module and the MPDIoU loss function are designed to reduce information redundancy in the model’s feature extraction, thereby improving the model’s detection accuracy. Liu et al. [23] incorporated a contrast enhancement module and a spatial information learning module into the Vision Transformer (ViT) [15], targeting foreground-background separation and complex posture recognition under different viewpoints, significantly enhancing the model’s accuracy and generalization performance in pest identification. Although Transformer-based models have achieved some success, they still exhibit limitations in fine-grained feature extraction. The self-attention mechanism in the Transformer tends to focus on globally salient regions (e.g., the main body contours of pests), making it difficult to capture locally discriminative details (e.g., microscopic texture differences in the wing veins of lepidopteran pests), which may result in a decrease in the performance of Transformer-based models.

Considering that CNN can effectively extract local features and spatial structural information from images, while Transformer can deeply mine long-range dependence relationships between different regions in images, some researchers have attempted to combine the two for crop pest classification. For example, Liu et al. [24] introduced a self-supervised Transformer based on a feature relation conditional filtering algorithm (FRCF) and a latent semantic masking autoencoder (LSMAE) for pest classification. Experimental results indicate that this approach outperforms CNN-based methods in the corresponding tasks. Peng et al. [25] developed a simplified yet effective framework for pest classification that extracts representative features using CNNs and employs a Transformer-based attention classifier to exploit spatial information, thereby improving the model’s performance. Hechen et al. [26] proposed a novel visual Transformer (DWViT-ES) that integrates an efficient suppressed self-attention mechanism and dilated windows. This model extends the effective receptive field of self-attention in the Visual Transformer and reduces information loss, while simplifying consecutive linear transformations, resulting in fewer parameters and lower computational cost without compromising accuracy. Although the above CNN-Transformer hybrid models have made some progress in crop pest classification, they still suffer from the following limitations. On the one hand, existing hybrid models leverage CNN for feature extraction, but struggle to effectively cope with the practical challenges of small or diverse pests. On the other hand, the Transformer encoder in existing hybrid models neglects the feature interactions among adjacent tokens, thus failing to effectively capture discriminative features of pests. Therefore, a new crop pest classification model (CT-PestNet) is proposed, and the main contributions are as follows:

1. A cross-scale feature distillation enhancement module (CFDE) is designed to respond to the challenges of diverse pest morphologies (with varying sizes and shapes) and complex backgrounds. The CFDE employs the “distillation-then-enhancement” strategy to enhance the model’s perception of small or diverse pests, thereby improving its classification accuracy and robustness in complex backgrounds.
2. A local token interaction module (LTIR) is constructed to overcome the insufficient modeling of adjacent token features in the Transformer encoder. The LTIR can achieve deep discriminative feature mining by enhancing the feature interaction among adjacent tokens, thereby improving the model’s processing ability and recognition performance for complex images.
3. Extensive experiments are conducted on the IP102 and D0 datasets. Experimental results demonstrate that the proposed model outperforms most existing intelligent crop pest classification models, fully demonstrating its effectiveness.

## Related theories

### Multi-scale feature extraction mechanisms

In computer vision, traditional single-scale feature extraction methods often struggle to simultaneously capture fine-grained local details and high-level semantic information of images. To address this issue, researchers have gradually developed a series of multi-scale feature extraction mechanisms that enable models to better adapt to the diversity of target objects to be classified (e.g., morphology, size, or structure) and effectively cope with complex and varying background environments.

For example, Luo et al. [27] pointed out that the discriminative features of leaf diseases in complex environments are often not sufficiently prominent, and single-scale feature extraction methods are prone to losing critical information. Therefore, they proposed a multi-scale feature fusion strategy to reduce information loss, thereby enhancing the model’s ability to classify leaf diseases in complex environments. Wang et al. [28] designed a multi-scale interactive information extraction module (MIIE), which not only captures richer multi-scale features from images but also maintains a relatively small parameter scale, outperforming traditional single-scale feature extraction methods. Luo et al. [29] introduced a multi-scale attention fusion module (MSAF), which can flexibly integrate features of different scales through an adaptive weighting mechanism, thereby improving the model’s ability to perceive and model subtle differences. Zhang et al. [30] developed a novel multi-scale feature fusion strategy (MSF) to address the limitations of insufficient feature extraction and inadequate feature fusion in existing image classification models. This strategy can efficiently integrate shallow detail information with deep semantic features, thereby improving the recognition ability of the model. Gao et al. [31] developed a multi-scale spatial Mamba module (MSpa-Mamba), which reduces computational burden and suppresses feature redundancy across multiple scanning paths through the multi-scale decomposition strategy, achieving efficient and precise spatial feature modeling.

Although the existing series of multi-scale feature extraction mechanisms can capture information at different scales from images, their feature fusion methods often rely on two operations: channel concatenation or element-wise addition. However, channel concatenation significantly increases the dimensionality of feature maps, leading to an increase in the computational complexity of the model; element-wise addition ignores the inherent differences between shallow texture details and deep semantic features, thus diluting fine‐grained discriminative features that are crucial in complex agricultural backgrounds. Therefore, a cross-scale feature distillation enhancement module (CFDE) is designed, which adopts a “distillation‑then‑enhancement” paradigm to filter out redundant information and background noise in multi-scale features and adaptively enhance the discriminative features. This design reduces computational overhead while simultaneously improving the model’s perception for small or diverse pests.

### Transformer encoder improvement strategies

To overcome the representation limitations of CNN caused by the local receptive field in computer vision, Dosovitskiy et al. proposed the ViT, which has made breakthrough progress in multiple image tasks by fully leveraging the advantages of the Transformer in global information modeling. Since the emergence of ViT, numerous researchers have focused on improving its core component (Transformer encoder) with the aim of further enhancing its performance.

For example, Zhou et al. [32] pointed out that as the network depth increases, the self-attention mechanism in ViT struggles to learn effective feature representations suitable for the current task. To address this issue, they proposed a Re-attention mechanism that successfully achieved deeper ViT training and performance improvement with almost no increase in computational overhead. Song et al. [33] argued that the self-attention mechanism in ViT requires substantial computational resources. Therefore, they introduced a novel self-attention mechanism that significantly reduces the computational complexity of the model and improves its linear scalability. At the same time, their model performs comparably to existing state-of-the-art nonlinear vision transformer models in multiple vision tasks. Xia et al. [34] abandoned the traditional fixed grid attention mechanism in ViT and designed a deformable attention mechanism (DAM) to enhance the model’s ability to capture key image features flexibly. The DAM is not limited by fixed positions and can adaptively adjust attention regions based on the input content, thereby more effectively extracting the morphological and structural features of objects. Patel et al. [35] developed a multi-resolution overlapping attention module (MOA) to efficiently transfer neighborhood pixel information, addressing the shortcomings of the local Transformer [36] in spatial global information aggregation. Numerous experiments on datasets such as CIFAR and ImageNet-1K have shown that their method outperforms previous visual transformers and has a relatively small number of parameters. Zhang et al. [37] constructed a Transformer-based pest detection framework. In this framework, a fusion step attention mechanism was designed, and the cross-entropy loss function was improved, significantly enhancing the model’s ability to detect and locate small target pests in complex backgrounds. Saranya et al. [38] claimed that the traditional attention mechanism in ViT neglects the spatial correlations between image patches. To overcome this, they proposed a hybrid pooling multi-head attention (HPMA) mechanism to strengthen discriminative features and suppress irrelevant information, improving the model’s performance in fine-grained feature capture and long-range dependency modeling.

Although the existing series of Transformer encoder improvement strategies can enhance the performance of ViT to some extent, most of them primarily focus on global context scaling, sparse attention distribution, or content‑adaptive region mapping. Moreover, the above strategies all process serialized image patches through highly homogeneous global matrix projections, ignoring the feature interactions among adjacent tokens, which hinders the deep extraction of discriminative features of pests. Therefore, a local token interaction module (LTIR) is proposed, which can achieve the deep mining of discriminative features of pests by enhancing the feature interaction among adjacent tokens, thereby improving the model’s processing ability and recognition performance for complex images.

## Proposed method

### Overall architecture

**Fig 1:**
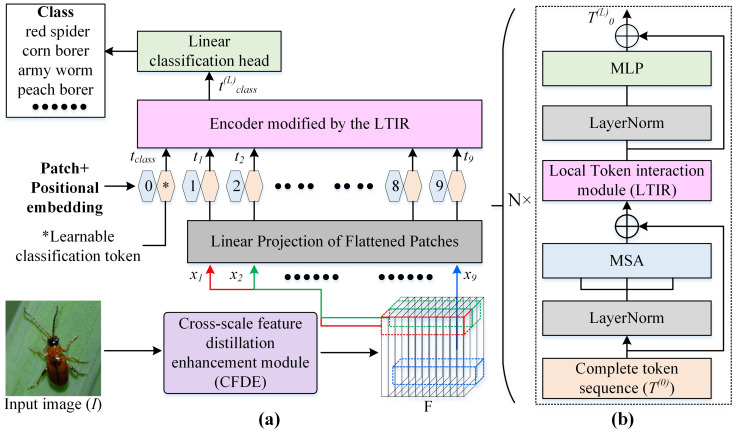
(a) Overall framework of the proposed crop pest classification model (CT-PestNet). (b) Internal architecture of the encoder modified by the local token interaction module (LTIR).

As illustrated in Fig 1(a), the proposed CT-PestNet mainly consists of two parts: the cross-scale feature distillation enhancement module (CFDE) and the ViT with an encoder modified by the local token interaction module (LTIR). Specifically, given an input image *I*, the CFDE first extracts the feature map *F* with multi-scale pest-discriminative perception ability, which is formulated as:


$$\begin{array}{c} F\in {\mathbb{R}}^{63\times 63\times 48}=CFDE(I) \end{array}$$ (1)

Next, *F* is partitioned into 9 non-overlapping patches of fixed size, and which are flattened into vectors. The mathematical expressions are:


$$\begin{array}{c} P_{n}\in {\mathbb{R}}^{21\times 21\times 48},n=1,2,...,9,\;x_{n}=vec(P_{n})\in {\mathbb{R}}^{21168} \end{array}$$ (2)

where $P_{n}$ and $x_{n}$ denote the *n*-th patch and its corresponding feature vector, respectively. Then, $x_{n}$ is mapped into a *D*-dimensional token vector via a learnable linear projection layer (Linear Projection of Flattened Patches). In this work, we set *D* = 1024, and the process is formulated as:


$$\begin{array}{c} t_{n}=W_{p}\cdot x_{n}+e_{n}^{\left(pos\right)} \end{array}$$ (3)

Here, $W_{p}\in {\mathbb{R}}^{D\times 21168}$ is the linear projection matrix, and $e_{n}^{\left(pos\right)}\in {\mathbb{R}}^{D}$ is the learnable positional embedding corresponding to the *n*-th patch. Meanwhile, a learnable classification token $t_{class}\in {\mathbb{R}}^{D}$ is prepended to the beginning of $t=[t_{1},t_{2},...,t_{9}{]}^{⊤}\in {\mathbb{R}}^{9\times D}$ to form the complete token sequence $T^{\left(0\right)}=[t_{class},t_{1},t_{2},...,t_{9}{]}^{⊤}\in {\mathbb{R}}^{10\times D}.$ Subsequently, *T*^(0)^ is processed by L stacked encoders modified by LTIR. The calculation process can be described as follows:


$$\begin{array}{c} T_{0}^{\left(L\right)}={Encoder}_{modified}^{LTIR}(T^{\left(0\right)}) \end{array}$$ (4)

Finally, the feature vector corresponding to the classification token is extracted from the final output sequence $T_{0}^{\left(L\right)}$, denoted as $t_{class}^{\left(L\right)}$, and fed into a linear classification head to perform category prediction.


$$\begin{array}{c} \hat{y}=Softmax(W_{class}\;t_{class}^{\left(L\right)}+b_{class}) \end{array}$$ (5)

Where $W_{class}$ and $b_{class}$ represent the weights and bias of the linear classification head, respectively, and $\hat{y}$ denotes the probability distribution of the model’s category prediction for the current pest image.

### Cross-scale feature distillation enhancement module (CFDE)

**Fig 2:**
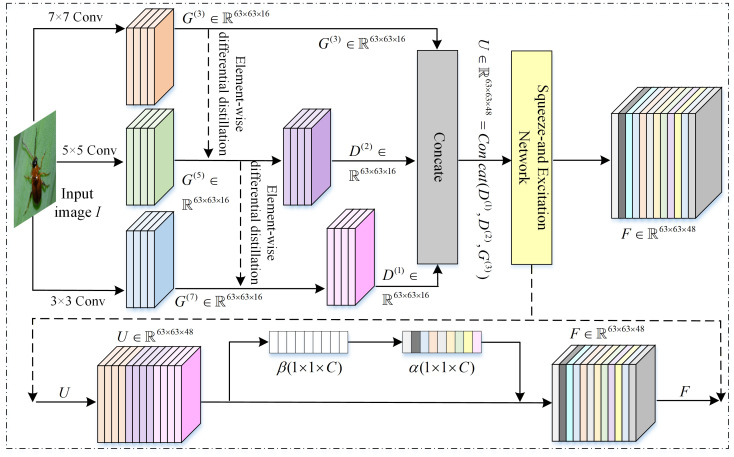
Specific structure of the cross-scale feature distillation enhancement module (CFDE).

As shown in Fig 2, the core principle of CFDE is to capture complementary features through parallel multi-scale convolutions, remove redundant information via feature distillation, and finally adaptively enhance task-relevant discriminative features by an attention mechanism. Specifically, given an input image *I*, three groups of convolutional operators with different kernel sizes are applied to *I*, producing three feature maps with varying granularities:


$$\begin{array}{c} G^{\left(7\right)}=\phi_{7}(I)\in {\mathbb{R}}^{63\times 63\times 16},\;G^{\left(5\right)}=\phi_{5}(I)\in {\mathbb{R}}^{63\times 63\times 16},\;G^{\left(3\right)}=\phi_{3}(I)\in {\mathbb{R}}^{63\times 63\times 16} \end{array}$$ (6)

Here, $\phi_{k}$ denotes the convolution operation with a kernel size k×k. Considering that responses of different kernels are correlated (larger kernels often contain local texture information captured by smaller kernels), to suppress redundant information, this paper performs element-wise differential distillation on adjacent-scale feature maps:


$$\begin{array}{c} D^{\left(1\right)}\in {\mathbb{R}}^{63\times 63\times 16}=G^{\left(7\right)}⊖G^{\left(5\right)},\;D^{\left(2\right)}\in {\mathbb{R}}^{63\times 63\times 16}=G^{\left(5\right)}⊖G^{\left(3\right)} \end{array}$$ (7)

where ⊖ denotes element-wise subtraction. Intuitively, *D*^(1)^ highlights “newly added structural information under a larger receptive field,” while *D*^(2)^ emphasizes “morphological contours newly introduced by the medium scale relative to the small scale.” In this way, the scale-gain information can be separated, thereby reducing the computational waste caused by repeatedly feeding redundant information. The distilled features *D*^(1)^, *D*^(2)^ are concatenated with *G*^(3)^ along the channel dimension, followed by a Squeeze-and-Excitation Network (SENet) to adaptively recalibrate channel-wise feature responses:


$$\begin{array}{c} U\in {\mathbb{R}}^{63\times 63\times 48}=Concat(D^{\left(1\right)},D^{\left(2\right)},G^{\left(3\right)}) \end{array}$$ (8)

$$\begin{array}{c} \beta \in {\mathbb{R}}^{48}=\frac{1}{63\times 63}\sum_{i=1}^{63}\sum_{j=1}^{63}U(i,j),\;\alpha \in {\mathbb{R}}^{48}=\sigma (W_{2}\cdot \delta (W_{1}\cdot \beta )) \end{array}$$ (9)

where β is the channel-wise statistical descriptor; *W*_1_ and *W*_2_ are the weights of the two fully connected layers in SENet; *r* is the reduction ratio (set to 4 in this work); δ and σ denote the ReLU and Sigmoid activation functions, respectively. Finally, the feature map *U* is recalibrated using the attention weights α: the learned weight α is used to reweight *U* channel-wise, yielding the enhanced output feature map *F*:


$$\begin{array}{c} F\in {\mathbb{R}}^{63\times 63\times 48}=\alpha ⊙U \end{array}$$ (10)

where ⊙ denotes channel-wise multiplication. *F* is the discriminative feature that contributes to the current pest identification task.

### Encoder modified by the local token interaction module (LTIR)

The essential idea of LTIR is to improve the structure of the encoder in ViT via an explicit local neighborhood interaction paradigm, aiming to mine deep discriminative features and enhance the processing capability and recognition performance for complex images.

**Fig 3:**
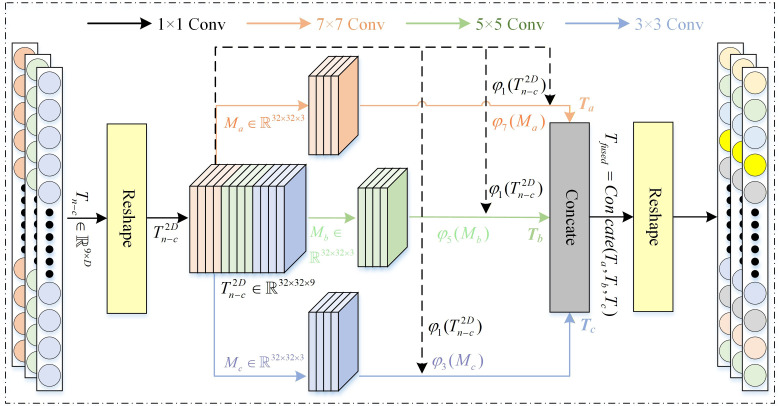
Implementation details of the local token interaction module (LTIR).

The structure of the encoder modified by LTIR is shown in Fig 1(b), which consists of multi-head self-attention (MSA), LTIR (as shown in Fig 3), and multi-layer perceptron (MLP), and applies layer normalization (LayerNorm) and residual connections. Specifically, given a complete token sequence $T^{\left(0\right)}=[t_{class},t_{1},t_{2},...,t_{9}{]}^{⊤}\in {\mathbb{R}}^{10\times D}$, it first passes through an LN layer followed by MSA, with a residual connection:


$$\begin{array}{c} \tilde{T}=MSA(LayerNorm(T^{\left(0\right)}))+T^{\left(0\right)} \end{array}$$ (11)

Here, MSA is defined as:


$$\begin{array}{cc} & MSA(Q,K,V)=Concat({head}_{1},...,{head}_{h})\;W^{O},\\ & {head}_{i}=Attention(QW_{i}^{Q},\;KW_{i}^{K},\;VW_{i}^{V}),\\ & Attention(Q,K,V)=softmax\;\left(\frac{QK^{⊤}}{\sqrt{d_{k}}}\right)V \end{array}$$ (12)

Next, the sequence $T_{n-c}\in {\mathbb{R}}^{9\times D}$ that excludes the classification token is separated from $\tilde{T}$ and reshaped into a two-dimensional feature map $T_{n-c}^{2D}$. To fully exploit interactions among neighboring tokens, $T_{n-c}^{2D}$ is split into three groups of feature maps along the channel dimension: $M_{a},M_{b},M_{c}$. Meanwhile, different-scale convolutions are applied to these feature maps, which are element-wise added with a channel-reduced feature map obtained by a 1×1 convolution on $T_{n-c}^{2D}$. Subsequently, the resulting feature maps from different branches are concatenated along the channel dimension to obtain the final feature map $T_{fused}$. Such design can achieve feature interaction by blending spatial neighborhood information and intra-group channel features. The mathematical formulation is:


$$\begin{array}{c} T_{n-c}^{2D}\in {\mathbb{R}}^{32\times 32\times 9}=Reshape((T_{n-c}{)}^{⊤})=Concat(M_{a},M_{b},M_{c}),\;M_{a},M_{b},M_{c}\in {\mathbb{R}}^{32\times 32\times 3} \end{array}$$ (13)

$$\begin{array}{c} T_{a}=\phi_{7}(M_{a})⊕\phi_{1}(T_{n-c}^{2D}),\;T_{b}=\phi_{5}(M_{b})⊕\phi_{1}(T_{n-c}^{2D}),\;T_{c}=\phi_{3}(M_{c})⊕\phi_{1}(T_{n-c}^{2D}) \end{array}$$ (14)

$$\begin{array}{c} T_{fused}=Concat(T_{a},T_{b},T_{c}) \end{array}$$ (15)

Here, each channel of *M* corresponds to a token, ⊕ denotes element-wise addition. Finally, $T_{fused}$ is reshaped back to a token sequence (with the same dimensionality as the input), prepended with the classification token, and then fed into the subsequent MLP, yielding the final output sequence $T_{0}^{\left(L\right)}$ of the *L*-th encoder layer.

### Ethics statement

Both the IP102 dataset and the D0 dataset used in the experiments and analysis section are two publicly available datasets that are widely adopted in crop pest recognition. The IP102 dataset can be downloaded from https://github.com/xpwu95/IP102, while the D0 dataset is available at https://github.com/ratulMahjabin/D0-dataset. According to the original dataset descriptions, the images were collected from open-field agricultural environments and web sources by the dataset authors. In this study, we did not conduct any additional web scraping or field collection. Furthermore, our use of these datasets is confined to non-commercial, scientific research purposes, and we ensure full compliance with the access conditions provided by the original data sources.

### Experiments and analysis

To validate the effectiveness and generalization capability of the proposed CT-PestNet, extensive experiments were conducted and evaluated on two publicly available crop pest datasets: IP102 and D0. To ensure the reliability of our findings, each experiment was independently conducted five times, with all results expressed as mean±standard deviation. The computational environment consisted of a Windows 10 workstation equipped with a 13th Gen Intel^®^ Core^TM^ i5-13400F processor and an NVIDIA GeForce RTX 3080 graphics card. Regarding the software implementation, all codes were written in Python, with PyTorch serving as the deep learning backbone and PyCharm functioning as the integrated development environment.

### Dataset description

**Fig 4:**
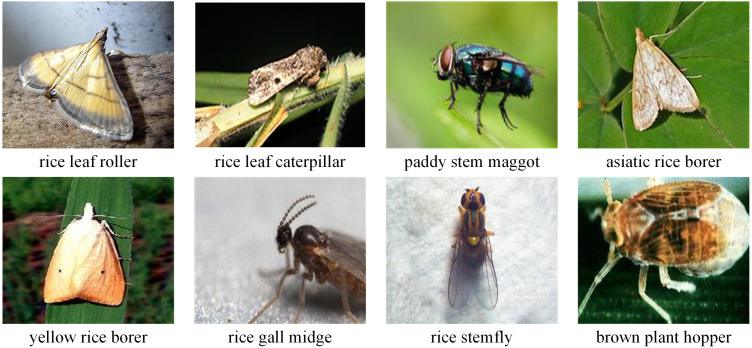
Example images of some pest categories from the IP102 dataset.

The IP102 dataset [39] is a large-scale benchmark dataset for pest classification, which is primarily composed of images collected from various search engines and entomology-related websites. The dataset encompasses 102 distinct crop pest categories, with a total of 75,222 images. The image scenes in the dataset are realistic, with complex and varied backgrounds, varying lighting conditions, diverse shooting angles, and some images containing noise or watermarks, highly reproducing the actual monitoring environment in the field. Fig 4 presents example images of some categories in the dataset. In this study, the IP102 dataset was divided into three parts: 45,095 images for training, 7,508 images for validation, and 22,619 images for testing.

**Fig 5:**
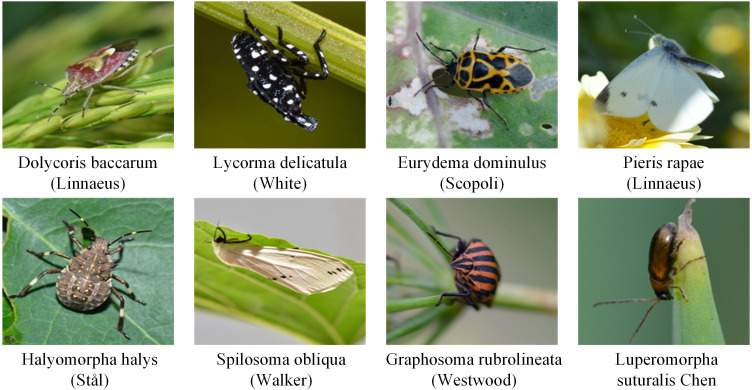
Example images of some pest categories from the D0 dataset.

The D0 dataset [40] is a relatively smaller-scale but high-quality field pest dataset, containing 4,508 images across 40 pest categories. Examples of some categories in this dataset are shown in Fig 5. In this study, the dataset was divided into three subsets: training set, validation set, and testing set. The classification method is as follows: for each category, 70% of the samples are randomly selected for training, and the remaining 30% are used for validation and testing in a 3:7 ratio.

### Performance comparison with classic mainstream models

**Fig 6:**
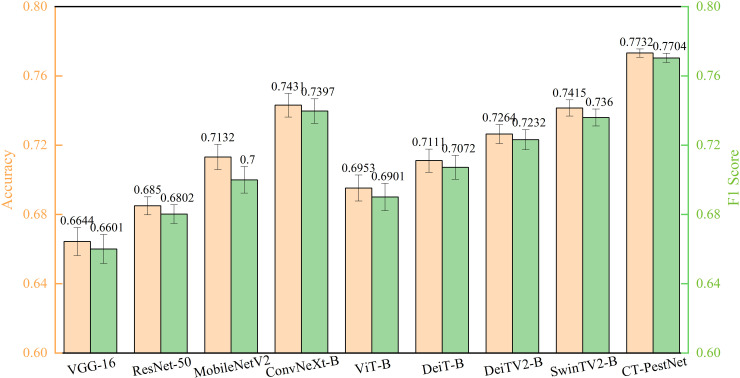
Performance comparison (Accuracy and F1-Score) between CT-PestNet and classic mainstream models (CNN-based and Transformer-based models) on the IP102 dataset.

**Fig 7:**
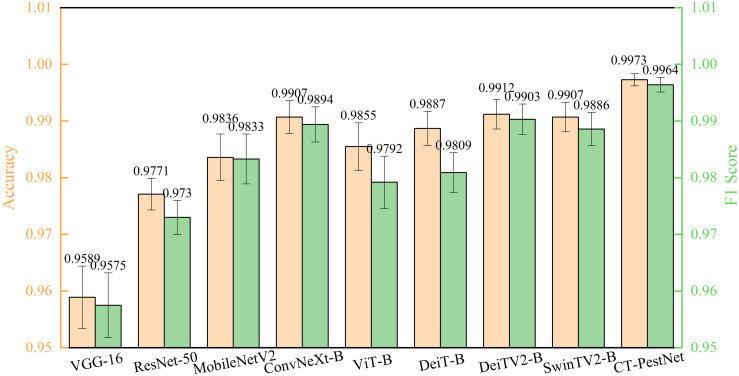
Performance comparison (Accuracy and F1-Score) between CT-PestNet and classic mainstream models (CNN-based and Transformer-based models) on the D0 dataset.

To verify the performance of CT-PestNet, it is compared with some classic mainstream models widely recognized in computer vision, including CNN-based models VGG-16 [7], ResNet-50 [8], MobileNetV2 [9], ConvNeXt-B [10], as well as Transformer-based models ViT-B [17], DeiT-B [18], DeiTV2-B [19], and Swin TransformerV2-B (SwinTV2-B) [20]. The results are shown in Fig 6 (IP102 dataset) and Fig 7 (D0 dataset).

From Fig 6, it can be seen that on the large-scale IP102 dataset with complex backgrounds and numerous categories, the accuracy (77.32%) and F1 score (77.04%) of CT-PestNet significantly surpass all comparison models. Specifically, compared to the CNN-based model ConvNeXt-B, which has the best performance, CT-PestNet has improved accuracy and F1 score by 3.01% and 3.07%, respectively; compared to the best-performing Transformer-based model SwinTV2-B, CT-PestNet has improved accuracy and F1 score by 3.17% and 3.44%, respectively. This result indicates that relying solely on traditional CNNs for local feature extraction or standard Transformers for global relationship modeling has limitations. On the contrary, CT-PestNet can leverage the advantages of CNN in local detail feature extraction and Transformer in modeling between distant regions, thereby achieving better performance.

As shown in Fig 7, all models achieved high performance on the D0 dataset, which can be attributed to its relatively simple background and more pronounced inter-class differences. Nevertheless, CT-PestNet still attains the best results, achieving 99.73% accuracy and 99.64% F1-score, respectively, surpassing other powerful contenders such as ConvNeXt-B (99.07%/98.94%) and DeiTV2-B (99.12%/99.03%). This proves that even on relatively simple tasks, the CFDE and LTIR modules do not have negative effects, but can further improve the classification accuracy and stability of the model through more refined feature processing mechanisms.

**Fig 8:**
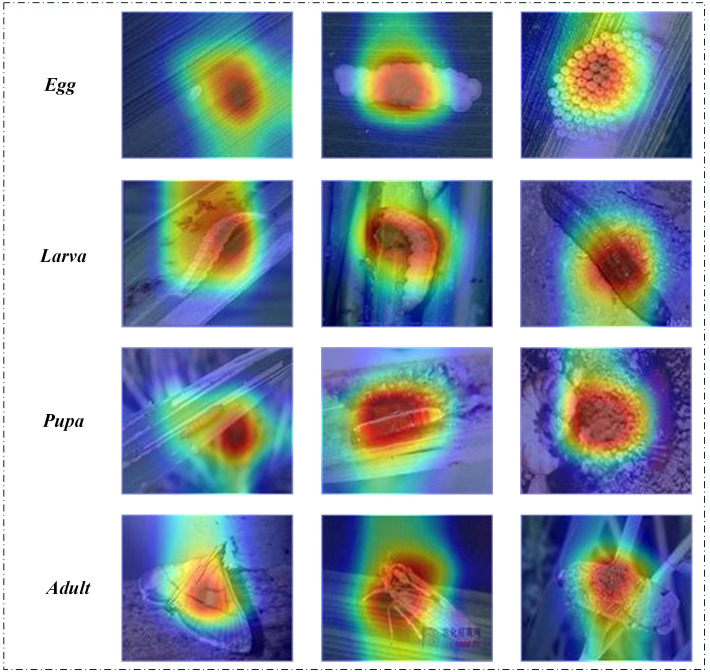
Grad-CAM visualization results of CT-PestNet for some samples from the IP102 dataset.

**Fig 9:**
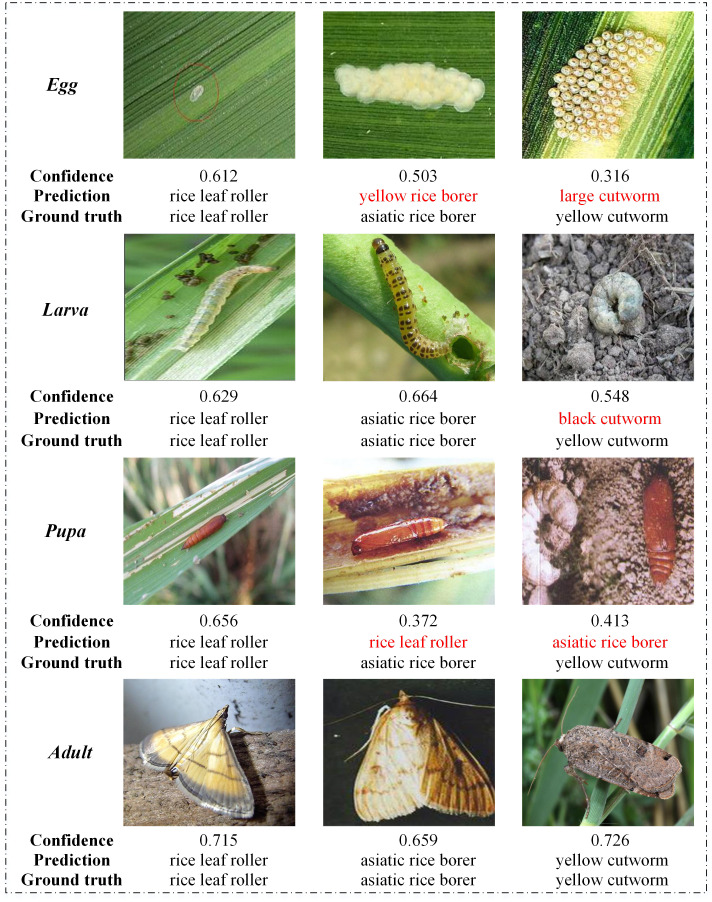
Prediction results of CT-PestNet for some samples from the IP102 dataset.

**Fig 10:**
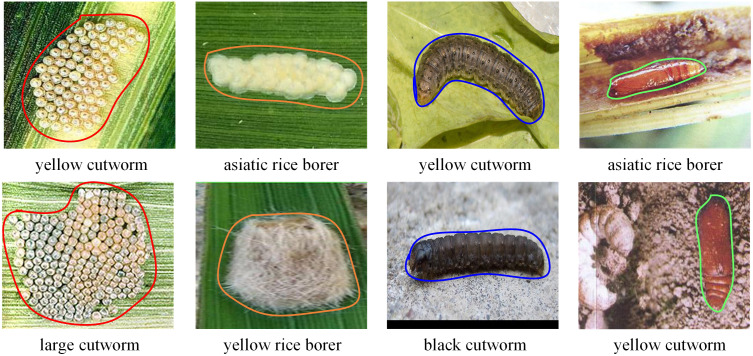
Representative misclassified samples exhibiting high morphological similarity within the same life stage on the IP102 dataset.

To gain deeper insight into the discriminative basis and error sources of the proposed CT-PestNet in the task of crop pest classification, this paper presents the model’s Grad-CAM visualization, prediction results, and morphological similarity analysis of misclassified samples for selected examples from the IP102 dataset, as shown in Fig 8, Fig 9, and Fig 10, respectively. These three sets of results elucidate the effectiveness and limitations of CT-PestNet from three progressive perspectives: “model attention regions—prediction performance—root causes of misclassification.” As can be observed from the Grad-CAM visualization results in Fig 8, CT-PestNet can accurately focus on the pest body regions in most samples, rather than dispersedly responding to background textures over large areas. This indicates that the proposed CFDE and LTIR modules can, to a certain extent, suppress background interference and guide the model to learn more discriminative target representations. Specifically, across the four life stages of Egg, Larva, Pupa, and Adult, the high-response regions of the heatmaps are predominantly concentrated on the target insect bodies or egg masses themselves, while the responses to surrounding elements such as leaf textures, branch backgrounds, and complex environmental areas are relatively weak. This suggests that CT-PestNet does not rely solely on scene context for classification but is capable of prioritizing key morphological regions related to pest categories during the classification process.

Examining the prediction results in Fig 9 reveals that, although the model generally possesses good localization and recognition capabilities, its performance varies significantly across different life cycle stages. Specifically, CT-PestNet achieves relatively accurate predictions with high confidence for the Adult stage. In contrast, more pronounced misclassifications are observed for the Egg, Larva, and Pupa stages. This indicates that while the model can localize the target regions in these samples, “correct localization” does not necessarily equate to “correct classification.” The reasons for this phenomenon are primarily twofold. First, compared to the adult stage, the number of samples for the egg, larval, and pupal stages is considerably smaller. This imbalance predisposes the model during training to learn the salient features of the more abundant categories or stages, making it difficult to establish stable representations for the minority stages, thereby reducing its generalization capability for pests in these stages. Second, for different pest species within the same life cycle stage, their macro-morphologies are often highly similar, with discriminative differences existing only in minute local regions. Such subtle differences are precisely the ones susceptible to variations in shooting angle, lighting conditions, image resolution, and background noise.

The misclassification phenomena observed in Fig 9 are further corroborated in Fig 10. Fig 10 presents several misidentified samples alongside their morphologically similar counterparts. Taking egg-stage samples as an example, the egg masses of different species all appear as light-colored, densely packed small granular structures, with primary differences lying only in aggregation patterns, arrangement density, or the substrate they attach to. For larval samples, different species are highly similar in body shape, coloration, and segmentation, with distinctions confined to subtle variations in local textures and integument details. For pupal samples, the targets typically present as brownish, fusiform or cylindrical structures, making them even more challenging to distinguish visually.

Synthesizing the results from Fig 8, Fig 9, and Fig 10, it can be concluded that the proposed CT-PestNet can accurately attend to pest subjects under complex background conditions and demonstrates strong classification capability for the adult stage. This confirms its distinct advantages in target localization and discriminative feature extraction. However, the misclassification of egg, larval, and pupal stage samples highlights that data imbalance and high inter-class similarity remain core challenges in fine-grained crop pest classification tasks. This further underscores the need for future improvements at both the data and model levels. Potential strategies include stage-balanced sampling, fine-grained local feature constraints, class prototype learning, or metric learning to further enhance the model’s discriminative ability for pests in their early growth stages.

### Performance comparison with state-of-the-art methods

To further evaluate the effectiveness of CT-PestNet, it is compared with some state-of-the-art models designed specifically for pest classification tasks published in recent years, including AM-ResNet [13], PCNet [14], IRNV2 [15], VRFNet [16], MNV2 [39], FRCF+LSMAE [24], C-ViT [25], DWVit-ES [26], and Pest-ConFormer [40]. The results are summarized in Table 1 (IP102 dataset) and Table 2 (D0 dataset).

**Table 1:** Performance comparison (Accuracy and F1-Score) between CT-PestNet and state-of-the-art methods on the IP102 dataset.

Method	Category	Accuracy (%)	F1 Score (%)
AM-ResNet	CNN-based models	72.99±0.65	–
PCNet		73.70±0.58	–
IRNV2		71.84±0.71	64.06±0.76
VRFNet		68.34±0.79	68.34±0.74
FRCF+LSMAE	Transformer-based models	74.69±0.47	74.36±0.51
C-ViT		74.89±0.43	–
DWVit-ES		76.00±0.38	–
CT-PestNet (Ours)	–	77.32±0.24	77.04±0.27

As shown in Table 1, on the IP102 dataset, CT-PestNet outperforms all comparison models with an accuracy of 77.32% and an F1 score of 77.04%. Specifically, compared with PCNet, which performs the best in existing CNN-based models, and DWVit-ES, which performs the best in existing Transformer-based models, CT-PestNet has improved the accuracy by 3.62% and 1.32%, respectively. This result further indicates that the performance improvement of CT-PestNet is not a random benefit brought by the “stacked model scale,” but stems from two key mechanisms designed for the difficulties of pest images (small targets, subtle morphological differences, and complex backgrounds): CFDE (removing redundancy first and then strengthening) can effectively reduce the repeated responses between features of different scales, making the model more focused on the “new information brought by scale differences”; LTIR can strengthen the fine-grained feature correlation between adjacent regions, achieving a more comprehensive and robust feature representation.

It is not difficult to see from Table 2 that CT-PestNet performs equally well on the D0 dataset, with an accuracy and F1 score of 99.73% and 99.64%, respectively. Although the improvement is relatively small in numerical terms compared to other models, considering that the performance of all models on the D0 dataset is close to the upper limit, this improvement is still meaningful. The results indicate that CT-PestNet can still reduce misclassification on a few hard samples under almost saturated accuracy, thereby improving overall stability and reliability. Overall, it indicates that even under different data characteristics, both the proposed CFDE and LTIR modules can play a positive role, enabling CT-PestNet to exhibit strong generalization ability and task adaptability.

**Table 2:** Performance comparison (Accuracy and F1-Score) between CT-PestNet and state-of-the-art methods on the D0 dataset.

Method	Category	Accuracy (%)	F1 Score (%)
PCNet	CNN-based models	98.94±0.31	98.86±0.34
VRFNet		99.12±0.28	99.12±0.26
MNV2		99.65±0.18	99.28±0.21
FRCF+LSMAE	Transformer-based models	98.81±0.37	98.33±0.42
C-ViT		99.15±0.24	–
Pest-ConFormer		99.51±0.19	99.50±0.17
CT-PestNet (Ours)	–	99.73±0.11	99.64±0.13

Furthermore, to evaluate the computational efficiency of the proposed CT‑PestNet, its parameter count, FLOPs, and inference time are compared with several state‑of‑the‑art models, with the results summarized in Table 3. It can be observed that although CT‑PestNet incorporates both the CFDE and LTIR modules, its computational overhead remains competitive with existing state‑of‑the‑art models. Specifically, CT‑PestNet has 85.0M parameters, which is lower than VRFNet (88.6M), SwinT V2‑B (88.0M), and DWVit‑ES (91.0M), and comparable to C‑ViT (86.6M). Its FLOPs are 15.1G, lower than those of C‑ViT (17.6G) and DWVit‑ES (16.8G), and close to VRFNet and SwinT V2‑B (both 15.4G). In terms of inference time, CT‑PestNet takes 60.3 ms, which is slightly slower than C‑ViT (45.2 ms) but significantly faster than SwinT V2‑B (65.7 ms) and DWVit‑ES (83.6 ms). Moreover, although PCNet has the smallest parameter count (25.6M) and the fastest inference speed (12.5 ms), its classification accuracy is far inferior to that of CT‑PestNet, indicating that excessive pursuit of lightweight design compromises recognition accuracy. Overall, CT‑PestNet achieves the highest classification accuracy among all compared models while maintaining a reasonable level of computational resource consumption, confirming that the CFDE and LTIR modules enhance performance without introducing excessive additional computational burden.

**Table 3:** Computational efficiency comparison (Parameters, FLOPs, and Inference time) between CT-PestNet and several state-of-the-art models.

Method	Parameters (M)	FLOPs (G)	Inference time (ms)
PCNet	25.6M	4.1G	12.5 ms
VRFNet	88.6M	15.4G	28.6 ms
C-ViT	86.6M	17.6G	45.2 ms
SwinTV2-B	88.0M	15.4G	65.7 ms
DWVit-ES	91.0M	16.8G	83.6 ms
CT-PestNet (Ours)	85.0M	15.1G	60.3 ms

### Ablation study

**Fig 11:**
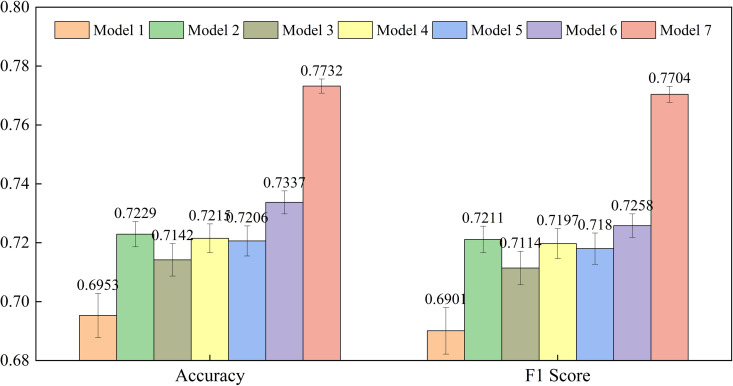
Ablation study results (Accuracy and F1-Score) on the IP102 dataset.

**Fig 12:**
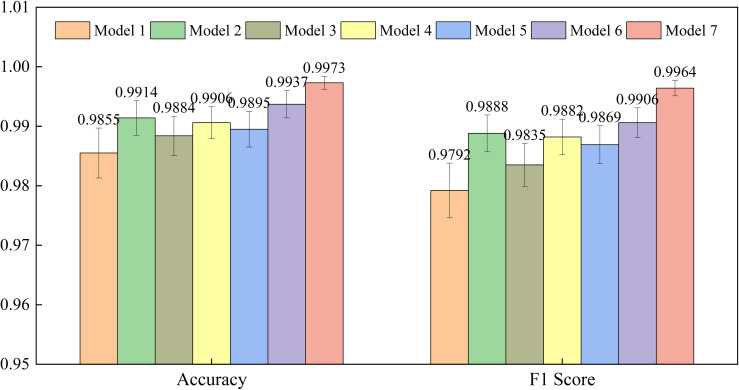
Ablation study results (Accuracy and F1-Score) on the D0 dataset.

To evaluate the effectiveness of the CFDE and LTIR modules and further verify the rationality of the internal structure design of CFDE, seven model variants are constructed: Model 1 (ViT-B), Model 2 (ViT-B+LTIR), Model 3 (ViT-B+CFDE (No SENet)), Model 4 (ViT-B+CFDE (1, 3, 5 Kernel)), Model 5 (ViT-B+CFDE (5, 7, 9 Kernel)), Model 6 (ViT-B+CFDE), and Model 7 (CT-PestNet (Ours)). The ablation results on the IP102 and D0 datasets are shown in Fig 11 and Fig 12, respectively.

As illustrated in Fig 11, Model 1 achieves an accuracy and F1-score of 69.53% and 69.01% on the IP102 dataset, respectively. When incorporating only LTIR (Model 2), the accuracy and F1-score increase to 72.29% and 72.11%, respectively; when incorporating only CFDE (Model 6), the accuracy and F1-score increase to 73.37% and 72.58%, respectively. Similarly, as shown in Fig 12, on the D0 dataset, Model 1 achieves an accuracy of 98.55% and an F1-score of 97.92%. Adding only LTIR (Model 2) improves the performance to 99.14% and 98.88%, while adding only CFDE (Model 6) improves it to 99.37% and 99.06%. These results indicate that CFDE can provide “cleaner and higher-information-density” input representations, while LTIR enhances local interactions, allowing fine-grained discriminative features to be better preserved and amplified in deeper representations.

Observing Model 3, Model 4, and Model 5 in Fig 11 and Fig 12, it can be found that when the SENet within CFDE is removed (Model 3), the accuracy drops to 71.42% on the IP102 dataset and 98.84% on the D0 dataset. Although its performance is still significantly better than the baseline (Model 1), it is noticeably inferior to CFDE integrated with the SENet (Model 6). This intuitively demonstrates that without SENet dynamically suppressing redundant information and enhancing discriminative features, CFDE cannot fully exploit its representational capacity. Meanwhile, when the convolution kernel combination (3, 5, 7) of CFDE is replaced with a smaller receptive field (1, 3, 5) (Model 4) or a larger receptive field (5, 7, 9) (Model 5), the model’s performance on both datasets similarly degrades to varying degrees. This phenomenon suggests that the original (7, 5, 3) multi-scale combination is optimal for capturing the rich contextual semantics of pest images, ranging from “large-scale structures to fine-grained details.” A kernel size that is too small results in the loss of essential contextual information from large receptive fields, whereas a kernel size that is too large may introduce excessive background noise, both of which disrupt the optimal feature distillation effect.

Furthermore, as observed in Fig 11 and Fig 12, when both LTIR and CFDE are incorporated simultaneously (Model 7), the proposed model achieves the highest accuracy and F1-score on IP102 and D0 datasets. This not only validates the effectiveness of each module but also demonstrates the strong complementarity between LTIR and CFDE. Together, they form a complete feature optimization pipeline from “macro-scale multi-scale perception” to “micro-scale local refinement,” thereby further driving the overall performance improvement of the model.

## Conclusion

This paper proposes a novel crop pest classification model (CT-PestNet). The proposed model can effectively integrate the advantages of CNNs and Transformers around the design concept of “removing redundancy before enhancing discrimination, and then mining local differences more deeply,” which is realized by the constructed cross-scale feature distillation enhancement module (CFDE) and local token interaction module (LTIR). Extensive experimental results on two publicly available datasets show that the performance of CT-PestNet is superior to some classic mainstream models based on CNNs or Transformers widely recognized in computer vision, as well as multiple existing state-of-the-art pest recognition methods, fully demonstrating the effectiveness of CT-PestNet in pest recognition tasks under the coexistence of complex backgrounds and fine-grained differences.

Although CT-PestNet achieves excellent performance, its efficacy is still highly correlated with the scale of labeled data. However, data annotation in the agricultural domain is costly and heavily dependent on expert knowledge. Therefore, future research could adopt semi-supervised learning paradigms to effectively exploit abundant unlabeled field images, thereby reducing the cost of manual annotation while further improving the model’s generalization ability in limited labeled data scenarios.
