# Supplementary material for: CT-PestNet: A novel crop pest classification model based on cross-scale feature distillation enhancement and vision transformer with local token interaction
Source: PLoS One. 2026 Aug 14;21(8):e0356061. doi: 10.1371/journal.pone.0356061 (PMC13475986; doi:10.1371/journal.pone.0356061)
Supplement: Supplementary file 1 [file pone.0356061.s002.docx]

**Original Manuscript ID:** PONE-D-26-13218

**Original Article Title:** “CT-PestNet: A Novel Crop Pest Classification Model Based on Cross-scale Feature Distillation Enhancement and Vision Transformer with Local Token Interaction”

Dear Editor,

Hello,

First, we would like to express our sincere gratitude to you and the peer-review team for the valuable feedback and meticulous review of our manuscript. Second, we have carefully considered the journal requirements and reviewers' comments and suggestions and made corresponding revisions to the full text to improve its quality and clarity. Finally, we believe these revisions will strengthen the academic quality and practicality of the manuscript. We have submitted the revised manuscript and a document detailing our point-by-point response to the journal requirements and reviewers' comments. We kindly ask the reviewers and the editorial team to review the revisions and hope for your approval.

Once again, we sincerely appreciate your time and effort, as well as your support and valuable feedback on our research.

Best regards,

<Youming Li> et al.

**Response to Comments from Editorial Office**

- **Specific Comments**

1. Please ensure that your manuscript meets PLOS ONE's style requirements, including those for file naming. The PLOS ONE style templates can be found at https://Journals.plos.org/plosone/s/file?id=wjVg/PLOSOne_formatting_sample_main_body.pdf and https://journals.plos.org/plosone/s/file?id=ba62/PLOSOne_formatting_sample_title_authors_affiliations.pdf.

**Author response:** We sincerely thank the editorial office for the reminder regarding the manuscript formatting requirements. In response to this concern, we have carefully reviewed the relevant guidelines on the PLOS ONE official website. Our manuscript was prepared using the LaTeX template available under the path: **Publish → Submission Guidelines → File Format → Read the LaTeX guidelines → Download the template** (<https://journals.plos.org/plosone/s/latex>). We have made every effort to ensure that the formatting aligns with the official template. We would greatly appreciate further instructions if there are any specific discrepancies we may have missed and will be happy to make the necessary adjustments.

2. Please note that PLOS ONE has specific guidelines on code sharing for submissions in which author-generated code underpins the findings in the manuscript. In these cases, we expect all author-generated code to be made available without restrictions upon publication of the work. Please review our guidelines at https://journals.plos.org/plosone/s/materials-and-software-sharing#loc-sharing-code and ensure that your code is shared in a way that follows best practice and facilitates reproducibility and reuse.

**Author response:** We sincerely thank the editorial office for the reminder regarding the code-sharing policy. We fully acknowledge and respect PLOS ONE’s emphasis on transparency and reproducibility in scientific research. However, we regret to inform you that the code developed for this study cannot be made publicly available due to institutional confidentiality agreements. The code is part of a project conducted under our institution’s internal regulations, prohibiting the public release of source code without prior approval.

We greatly appreciate your understanding, and we have done our best to describe the methodology in sufficient detail in the manuscript to ensure the reproducibility of our results. Should the editorial office require further clarification on specific technical aspects, we would happily provide additional information where possible.

3. We note that Figures 1, 2, 4, 5, 9 and 10 in your submission contain copyrighted images. All PLOS content is published under the Creative Commons Attribution License (CC BY 4.0), which means that the manuscript, images, and Supporting Information files will be freely available online, and any third party is permitted to access, download, copy, distribute, and use these materials in any way, even commercially, with proper attribution. For more information, see our copyright guidelines: http://journals.plos.org/plosone/s/licenses-and-copyright.

We require you to either (1) present written permission from the copyright holder to publish these figures specifically under the CC BY 4.0 license, or (2) remove the figures from your submission:

(1) You may seek permission from the original copyright holder of Figures 1, 2, 4, 5, 9 and 10 to publish the content specifically under the CC BY 4.0 license.

We recommend that you contact the original copyright holder with the Content Permission Form (http://journals.plos.org/plosone/s/file?id=7c09/content-permission-form.pdf) and the following text:

“I request permission for the open-access journal PLOS ONE to publish XXX under the Creative Commons Attribution License (CCAL) CC BY 4.0 (http://creativecommons.org/licenses/by/4.0/). Please be aware that this license allows unrestricted use and distribution, even commercially, by third parties. Please reply and provide explicit written permission to publish XXX under a CC BY license and complete the attached form.”

Please upload the completed Content Permission Form or other proof of granted permissions as an "Other" file with your submission.

In the figure caption of the copyrighted figure, please include the following text: “Reprinted from [ref] under a CC BY license, with permission from [name of publisher], original copyright [original copyright year].”

(2) If you are unable to obtain permission from the original copyright holder to publish these figures under the CC BY 4.0 license or if the copyright holder’s requirements are incompatible with the CC BY 4.0 license, please either i) remove the figure or ii) supply a replacement figure that complies with the CC BY 4.0 license. Please check copyright information on all replacement figures and update the figure caption with source information. If applicable, please specify in the figure caption text when a figure is similar but not identical to the original image and is therefore for illustrative purposes only.

**Author response:** We sincerely thank the editorial office for raising this important copyright compliance issue. We fully understand and respect PLOS ONE’s strict policy on open access under the CC BY 4.0 license. In response to your concerns regarding Figures 1, 2, 4, 5, 9, and 10, we have taken the following actions in our revised manuscript to ensure full compliance.

- **Replacement of the images to ensure compliance**

Both the IP102 dataset and the D0 dataset used in this study are two publicly available datasets that are widely adopted in crop pest recognition. The IP102 dataset can be downloaded from https://github.com/xpwu95/IP102, while the D0 dataset is available at https://github.com/ratulMahjabin/D0-dataset. Some of the original images in these datasets did contain third‑party logos or identifiers. In this revision, we have replaced all such original images with either logo‑free images from the same datasets or with our own pre‑processed versions that carry no third‑party marks. The new images are used solely for scientific illustration and do not include any commercial or copyrighted identifiers; thus, they fully meet the requirements of the CC BY 4.0 license.

- **Compliance with the academic use terms of the datasets**

We have strictly adhered to the official terms of use for these datasets, whose official page clearly states that “This dataset is free for academic usage”. Our use is exclusively for non‑commercial academic research, which is fully consistent with that condition.

- **New statement added to the revised manuscript**

As suggested by you and the reviewers, we have added an **“Ethics Statement”** section in the revised manuscript. In this section, we clearly state that all datasets used are publicly available academic datasets, and confirm our adherence to the data usage terms.

We believe that, with the above replacements and clarifications, all related images no longer raise third‑party copyright concerns and are fully suitable for publication in PLOS ONE. Thank you again for your time and professional guidance. We look forward to your feedback.

**Response to Comments from Reviewer #1**

- **Overall Assessment**

This paper presents a novel hybrid CNN-Transformer model for crop pest classification, with two interesting modules (CFDE for multi-scale feature distillation and LTIR for local token interaction). The experimental work is extensive and the results show clear improvements over several baselines. However, the manuscript has three major weaknesses that must be addressed before publication:

**Author response:** We sincerely thank you for the positive evaluation of our work and for the valuable comments. We truly appreciate your recognition of the novelty of our hybrid CNN-Transformer model, the comprehensiveness of our experiments, and the clear improvements over the baselines.

At the same time, we fully acknowledge and highly value your comments, which are extremely insightful and constructive, providing us with a clear direction for further improvement. In response, we have carefully revised and supplemented the manuscript. Regarding each specific comment you put forward, we have provided detailed responses one by one below. For your convenience in reviewing, we have used different colors to distinguish the content. Your valuable suggestions are highlighted in blue; our sincere recognition and gratitude for your comments are presented in black; and the supplementary explanations and specific revisions made to the content of the paper are all marked in red.

Thank you again for your constructive suggestions, which have helped us substantially improve the quality of the paper.

- **Specific Comments**

1. Add a statistical comparison table with p‑values (e.g., McNemar’s test) for the main accuracy/F1 differences.

**Author response:** We sincerely appreciate your constructive suggestion, which can greatly strengthen the credibility of our experimental results. To verify that the performance improvements of CT-PestNet are not coincidental, we conducted paired t-tests based on the Accuracy and F1-Score values obtained from five independent runs for all models. Specifically, for each baseline method, the corresponding results from the five runs were compared with those of CT-PestNet, and the resulting p-values are reported in Table Ⅰ and Table Ⅱ. Compared with McNemar’s test, which is mainly designed for paired sample-level correctness comparisons, the paired t-test is more suitable for evaluating repeated-run metric differences such as Accuracy and F1-Score in our experimental setting. All the p-values for Accuracy and F1-Score are shown in Table Ⅰ and Table Ⅱ below. It can be seen that all reported p-values are below 0.05, indicating that the improvements of CT-PestNet over the compared baseline methods are statistically significant.

**Table Ⅰ. Paired t-test p-values for pairwise performance comparisons of Accuracy and F1-Score on the IP102 dataset.**

| Category | Models | Methods | Accuracy P-value | F1-Score P-value |
| --- | --- | --- | --- | --- |
| Classic mainstream models  (**Fig 6**) | CNN-based models | VGG-16 | 0.0003 | 0.0007 |
|  |  | ResNet-50 | 0.0182 | 0.0235 |
|  |  | MobileNetV2 | 0.0012 | 0.0018 |
|  |  | ConvNeXt-B | 0.0049 | 0.0056 |
|  | Transformer-based models | ViT-B | 0.0008 | 0.0011 |
|  |  | DeiT-B | 0.0134 | 0.0158 |
|  |  | DeiTV2-B | 0.0385 | 0.0441 |
|  |  | SwinTV2-B | 0.0267 | 0.0315 |
| State-of-the-  art methods  (**Table 1**) | CNN-based  models | AM-ResNet | 0.0159 | 0.0172 |
|  |  | PCNet | 0.0214 | 0.0286 |
|  |  | IRNV2 | 0.0083 | 0.0097 |
|  |  | VRFNet | 0.0034 | 0.0042 |
|  | Transformer-based models | FRCF+LSMAE | 0.0452 | 0.0486 |
|  |  | C-ViT | 0.0321 | 0.0393 |
|  |  | DWVit-ES | 0.0408 | 0.0475 |

**Table Ⅱ. Paired t-test p-values for pairwise performance comparisons of Accuracy and F1-Score on the D0 dataset.**

| Category | Models | Methods | Accuracy P-value | F1-Score P-value |
| --- | --- | --- | --- | --- |
| Classic mainstream models  (**Fig 7**) | CNN-based models | VGG-16 | 0.0004 | 0.0012 |
|  |  | ResNet-50 | 0.0241 | 0.0265 |
|  |  | MobileNetV2 | 0.0025 | 0.0031 |
|  |  | ConvNeXt-B | 0.0068 | 0.0089 |
|  | Transformer-based models | ViT-B | 0.0017 | 0.0022 |
|  |  | DeiT-B | 0.0114 | 0.0147 |
|  |  | DeiTV2-B | 0.0358 | 0.0422 |
|  |  | SwinTV2-B | 0.0225 | 0.0291 |
| State-of-the-  art methods  (**Table 2**) | CNN-based  models | PCNet | 0.0193 | 0.0238 |
|  |  | VRFNet | 0.0147 | 0.0179 |
|  |  | MNV2 | 0.0421 | 0.0456 |
|  | Transformer-based models | FRCF+LSMAE | 0.0152 | 0.0185 |
|  |  | C-ViT | 0.0288 | 0.0342 |
|  |  | Pest-ConFormer | 0.0369 | 0.0431 |

We would like to sincerely clarify that, given that model performance metrics in the relevant literature are generally reported in the form of mean ± standard deviation, and due to the page layout and length limitations of the manuscript, we did not include the statistical significance testing tables (Table Ⅰ and Table Ⅱ) in the main text. We kindly ask for your understanding of our considerations. Naturally, if you deem it essential to present the table in the main text, we fully respect your opinion and will not hesitate to incorporate it accordingly.

2. Report results from at least 3 independent runs (mean ± std) for all models.

**Author response:** We fully appreciate your concern regarding the statistical reliability of our experimental results. We would like to sincerely apologize for only reporting the average values and inadvertently omitting the standard deviations in the initial draft. In fact, prior to receiving your valuable feedback, we had already conducted five independent complete runs for all models during our experiments. In this revised version, we have explicitly stated at the beginning of the "Experiments and analysis" section (**see page 8**) that all results are presented as "mean±standard deviation" derived from five independent runs. Furthermore, we have added corresponding error bars and standard deviation values to all experimental data in Figures 6, 7, 11, 12, as well as Tables 1 and 2, to intuitively demonstrate the stability of our models.

3. Add a table comparing model size (parameters), FLOPs, and inference time per image.

**Author response:** Thank you for raising this important point. Comparing model efficiency is indeed a crucial metric in practical applications. Following your suggestion, we have added a detailed comparison table (**Table 3**) at the end of the "Performance comparison with state-of-the-art methods" subsection (**see pages 11 and 12**). This table specifically showcases the number of parameters, FLOPs, and inference time for our model compared to the baselines, accompanied by a corresponding analysis of the experimental data.

4. Provide the source code and a brief README for reproducibility.

**Author response:** We completely understand the importance of open-source code for academic reproducibility. Regarding code sharing, we must honestly inform you that this study is part of an ongoing confidential project within our laboratory. Due to the internal management policies of our institution and research group, we are currently unable to release the full source code on public platforms without prior official approval. However, to fully address your concerns regarding reproducibility, our manuscript provides extremely detailed descriptions of the methodology, network architecture diagrams, and core mathematical formulas. If this is still insufficient for your understanding, we would be more than happy to privately provide you with a sanitized copy of the source code through unofficial channels solely for your review and evaluation. We kindly ask for your understanding regarding our institutional policy restrictions.

5. Undergo professional language editing.

**Author response:** Thank you very much for your attention to the language quality. Following your suggestion, we have carefully and comprehensively checked and refined the spelling, grammar, and sentence logic of the entire manuscript. Since the number of phrasing and syntactic adjustments made during this revision is substantial, and to keep the layout neat and easier for review, we have not used extensive yellow highlighting to indicate these modifications in the current revised manuscript. We realize this might cause some inconvenience during your evaluation, and we sincerely apologize for it in advance while kindly seeking your understanding.

6. Add an ethics statement regarding the use of insect images (even if publicly sourced).

**Author response:** Thank you for your rigorous oversight regarding research ethics. We have fully adopted your suggestion and added a brand new "Ethics Statement" section at the end of the revised manuscript (**see page 14**). This section elaborates on the legality of the data sources, copyright compliance, and related ethical declarations regarding our use of publicly available datasets.

7. Correct minor formatting issues (e.g., “Fig 1.(a)” → “Fig. 1(a)”, remove stray numbers on pages 17 and 9).

**Author response:** Thank you very much for your keen and detailed observations. We have corrected the specific formatting errors you pointed out. Furthermore, to prevent any residual issues of this kind, we conducted a comprehensive and systematic check of all figure captions, punctuation, and page number formats throughout the manuscript, ensuring that no similar irregularities remain in the revised manuscript.

We deeply appreciate your time and insightful comments, which have significantly improved the quality of our manuscript. We hope the above revisions and responses meet your expectations.

**Response to Comments from Reviewer #2**

- **Overall Assessment**

The manuscript PONE-D-26-13218, titled “CT-PestNet: A Novel Crop Pest Classification Model Based on Cross-scale Feature Distillation Enhancement and Vision Transformer with Local Token Interaction” presents a well-structured and technically sound deep learning framework that integrates CNN and Transformer architectures for crop pest classification. The proposed CT-PestNet model, along with CFDE and LTIR modules, reflects a thoughtful attempt to address both global and local feature extraction challenges. The experimental validation on benchmark datasets (IP102 and D0) is comprehensive, and the inclusion of visualization analyses (e.g., Grad-CAM) strengthens the interpretability of the results. Overall, the study demonstrates promising performance improvements and contributes to the growing literature on AI-driven agricultural monitoring. However, the manuscript requires revision before it can be considered for publication. The following comments are provided to improve clarity, rigor, and alignment with PLOS ONE standards:

**Author response:** We would like to express our sincere gratitude for your thorough review and constructive feedback on our manuscript. We are truly encouraged by your positive recognition of the overall design philosophy of our CT-PestNet model, the CFDE and LTIR modules, the comprehensive experimental validation on the IP102 and D0 datasets, as well as the interpretability enhancements provided by visualization analyses such as Grad-CAM.

At the same time, we fully appreciate and agree with your assessment that the manuscript still requires improvement in terms of clarity, rigor, and alignment with PLOS ONE’s standards. All the specific comments you have provided are immensely insightful and constructive, which offer a clear roadmap for strengthening our work. In response, we have thoroughly revised and supplemented the manuscript. Regarding each specific comment you put forward, we have provided detailed responses one by one below. For your convenience in reviewing, we have used different colors to distinguish the content. Your valuable suggestions are highlighted in blue; our sincere recognition and gratitude for your comments are presented in black; and the supplementary explanations and specific revisions made to the content of the paper are all marked in red.

We are deeply grateful for the time and expertise you have dedicated to helping us improve this study, and look forward to your further evaluation of our revised version.

- **Specific Comments**

1. The abstract is informative but could be improved by including quantitative performance metrics (e.g., accuracy gains) to better highlight the contribution. Currently, claims such as “outperforms most existing models” remain too general.

**Author response:** We sincerely appreciate your valuable suggestion on improving the abstract. We completely agree that specific data are far more convincing than general statements. According to your suggestion, we have specifically expanded the content at the end of the "Abstract" to clearly list the exact accuracy and F1 scores improvements of CT-PestNet compared with state-of-the-art models on both the IP102 and D0 datasets (**see page 1**). Now, readers can intuitively grasp the core performance advantages of our model upon reading the abstract.

2. Although limitations of CNN and Transformer models are mentioned, the precise research gap is not explicitly defined. It would be beneficial if the novelty of CFDE and LTIR is positioned more clearly against recent hybrid models.

**Author response:** Thank you for your keen observation regarding the lack of clarity in our research motivation. To highlight our innovation more effectively, we have expanded the 4th and 5th paragraphs of the "Introduction" section in the revised manuscript (**see page 3**). We not only deeply analyzed the limitations of existing hybrid models in handling multi-scale features and local details of pest images but also explicitly articulated how CFDE and LTIR specifically fill these research gaps. This makes the motivation of our proposed approach more rigorous and compelling.

3. The related work section primarily summarizes prior studies without critically analyzing their limitations. A comparative synthesis would strengthen the motivation for the proposed approach.

**Author response:** Thank you very much for pushing us to deepen our literature review. Following your suggestion, we have revisited the "Related Work" section. Specifically, we have added critical analysis paragraphs at the end of both the "Multi-scale Feature Extraction Mechanisms" and "Transformer Encoder Improvement Strategies" subsections (**see pages 4 and 5**), thereby logically highlighting the necessity and advantages of our proposed method.

4. Design decisions such as kernel sizes (7×7, 5×5, 3×3) and token dimensions (D = 1024) are presented without justification. A short rationale or sensitivity analysis is recommended.

**Author response:** Thank you for your attention to the design details of our model. Regarding the token dimension (D=1024), we would like to clarify that this parameter was strictly adopted from our baseline model, ViT-B, to ensure fairness and consistency in comparative experiments. As for the kernel sizes (7×7, 5×5, 3×3), this design was partly inspired by existing literature on multi-scale feature extraction. Furthermore, to verify the rationality of this selection, we have specifically added sensitivity comparison experiments in the "Ablation Study" subsection (**see pages 12 and 13**), and the results demonstrate that our selected (7×7, 5×5, 3×3) combination yields optimal performance in capturing multi-scale contextual information from pest images.

5. The manuscript reports accuracy and F1-score but lacks statistical significance testing or confidence intervals. It is suggested that variability across runs be reported.

**Author response:** We greatly appreciate your rigorous requirements regarding experimental statistical reliability. We would like to sincerely apologize for only reporting the average values and inadvertently omitting the standard deviations in the initial draft. In fact, prior to receiving your valuable feedback, we had already conducted five independent complete runs for all models during our experiments. In this revised version, we have explicitly stated at the beginning of the "Experiments and analysis" section (**see page 8**) that all results are presented as "mean±standard deviation" derived from five independent runs. Furthermore, we have added corresponding error bars and standard deviation values to all experimental data in Figures 6, 7, 11, 12, as well as Tables 1 and 2, to intuitively demonstrate the stability of our models.

Furthermore, to verify that the performance improvements of CT-PestNet are not coincidental, we conducted paired t-tests based on the Accuracy and F1-Score values obtained from five independent runs for all models. Specifically, for each baseline method, the corresponding results from the five runs were compared with those of CT-PestNet, and the resulting p-values are reported in Table Ⅰ and Table Ⅱ.

**Table Ⅰ. Paired t-test p-values for pairwise performance comparisons of Accuracy and F1-Score on the IP102 dataset.**

| Category | Models | Methods | Accuracy P-value | F1-Score P-value |
| --- | --- | --- | --- | --- |
| Classic mainstream models  (**Fig 6**) | CNN-based models | VGG-16 | 0.0003 | 0.0007 |
|  |  | ResNet-50 | 0.0182 | 0.0235 |
|  |  | MobileNetV2 | 0.0012 | 0.0018 |
|  |  | ConvNeXt-B | 0.0049 | 0.0056 |
|  | Transformer-based models | ViT-B | 0.0008 | 0.0011 |
|  |  | DeiT-B | 0.0134 | 0.0158 |
|  |  | DeiTV2-B | 0.0385 | 0.0441 |
|  |  | SwinTV2-B | 0.0267 | 0.0315 |
| State-of-the-  art methods  (**Table 1**) | CNN-based  models | AM-ResNet | 0.0159 | 0.0172 |
|  |  | PCNet | 0.0214 | 0.0286 |
|  |  | IRNV2 | 0.0083 | 0.0097 |
|  |  | VRFNet | 0.0034 | 0.0042 |
|  | Transformer-based models | FRCF+LSMAE | 0.0452 | 0.0486 |
|  |  | C-ViT | 0.0321 | 0.0393 |
|  |  | DWVit-ES | 0.0408 | 0.0475 |

**Table Ⅱ. Paired t-test p-values for pairwise performance comparisons of Accuracy and F1-Score on the D0 dataset.**

| Category | Models | Methods | Accuracy P-value | F1-Score P-value |
| --- | --- | --- | --- | --- |
| Classic mainstream models  (**Fig 7**) | CNN-based models | VGG-16 | 0.0004 | 0.0012 |
|  |  | ResNet-50 | 0.0241 | 0.0265 |
|  |  | MobileNetV2 | 0.0025 | 0.0031 |
|  |  | ConvNeXt-B | 0.0068 | 0.0089 |
|  | Transformer-based models | ViT-B | 0.0017 | 0.0022 |
|  |  | DeiT-B | 0.0114 | 0.0147 |
|  |  | DeiTV2-B | 0.0358 | 0.0422 |
|  |  | SwinTV2-B | 0.0225 | 0.0291 |
| State-of-the-  art methods  (**Table 2**) | CNN-based  models | PCNet | 0.0193 | 0.0238 |
|  |  | VRFNet | 0.0147 | 0.0179 |
|  |  | MNV2 | 0.0421 | 0.0456 |
|  | Transformer-based models | FRCF+LSMAE | 0.0152 | 0.0185 |
|  |  | C-ViT | 0.0288 | 0.0342 |
|  |  | Pest-ConFormer | 0.0369 | 0.0431 |

It can be seen that all reported p-values are below 0.05, indicating that the improvements of CT-PestNet over the compared baseline methods are statistically significant. However, we would like to sincerely clarify that, given that model performance metrics in the relevant literature are generally reported in the form of mean ± standard deviation, and due to the page layout and length limitations of the manuscript, we did not include the statistical significance testing tables (Table Ⅰ and Table Ⅱ) in the main text. We kindly ask for your understanding of our considerations. Naturally, if you deem it essential to present the table in the main text, we fully respect your opinion and will not hesitate to incorporate it accordingly.

6. Given the high performance on the D0 dataset (99.73% accuracy), concerns regarding overfitting should be discussed, particularly considering dataset size.

**Author response:** Thank you for raising this valid concern regarding the risk of overfitting. We would like to clarify that our model achieved favorable accuracy and F1-score on both the training and test sets of the D0 dataset, and the performance on the two sets was highly consistent. Therefore, the typical overfitting pattern, namely extremely high training performance accompanied by substantially degraded test performance, was not observed. This suggests that our model effectively captured discriminative features in the D0 dataset rather than merely memorizing the training samples. Based on our further analysis, this result may be related to the characteristics of the D0 dataset, which contains a relatively small number of pest categories, has a balanced class distribution, shows clear inter-class differences, and provides high-quality images with limited background interference.

7. The manuscript identifies class imbalance (e.g., fewer egg/larva samples), yet no mitigation strategy (e.g., reweighting, augmentation) is implemented. This should be addressed experimentally.

**Author response:** Thank you very much for accurately pointing out an area that was underdeveloped in our work. We candidly admit that while we identified the issue of class imbalance in pest categories in the manuscript, the core focus of this study was on proposing the CFDE and LTIR modules to improve basic feature extraction and local interaction. Consequently, our current work does not yet incorporate specific mitigation strategies for imbalance. However, following your constructive feedback, we have added some contents at the end of the "Performance comparison with classic mainstream models" subsection (**see page 10**). We not only clarify that data imbalance and strong inter-class similarity are core challenges in pest fine-grained classification but also specifically discuss possible strategies to address them. In our future work, we will further validate these strategies through systematic experiments and explore their effectiveness in improving the robustness and generalization ability of pest classification models.

8. While CFDE and LTIR contributions are evaluated, the study lacks component-level analysis (e.g., SENet contribution, kernel variation impact).

**Author response:** Thank you very much for this suggestion, which has made our experimental analysis more comprehensive. To deeply analyze the effectiveness of each component, we have significantly expanded the "Ablation Study" subsection (**see pages 12 and 13**) in the revised manuscript. We not only evaluated the presence or absence of CFDE and LTIR but also specifically designed variants to assess the contribution of SENet and the impact of different kernel sizes (1×1, 3×3, 5×5 and 5×5, 7×7, 9×9). These detailed ablation data and analyses provide strong evidence for the rationality of adopting the SENet and specific receptive field kernels as core components of our model.

9. Some figures (e.g., Fig 6–7) are referenced without detailed interpretation in the text. Figure captions should be more self-contained and explanatory. Picture of rice stem fly in Figure 4 seems licensed as logo shows on it, please use your own pictures of these pests otherwise get permission from the original sources. Same comment must be dealt with for all other such pictures.

**Author response:** Thank you very much for your careful scrutiny regarding figure formatting and copyright issues. In response to your suggestions, we have taken three specific measures:

First, we have thoroughly revised all figure titles and captions throughout the manuscript, ensuring that the captions not only present the data but also provide sufficient contextual information for readers to understand them independently. Second, regarding image copyright issues, we conducted a comprehensive self-examination. For Figure 4 and all other original images in the manuscript that might have previously contained third-party logos, we have replaced them with images of the same category without such identifiers, selected from the IP102 and D0 datasets. We also ensured that our entire experimental process strictly adhered to the academic usage terms of these datasets. Third, to completely eliminate copyright concerns and enhance transparency, we have added a new "Ethics Statement" section (**see page 14**) in the revised manuscript, explicitly declaring the legality of the data sources.

10. It is suggested that the authors cite recent relevant work to strengthen the manuscript’s broader context. In particular, Ghaffar et al. (2025) (https://doi.org/10.55627/pbiotech.003.04.1702) on AI-driven plant stress management and Zulkiffal et al. (2025) (https://doi.org/10.55627/pbiotech.003.04.1709) on data-driven wheat improvement for rust resistance provide useful complementary perspectives. Their inclusion would enhance the interdisciplinary relevance of the study.

**Author response:** Thank you very much for helping us broaden the academic perspective of our study. We completely agree that placing research within a broader interdisciplinary context significantly enhances the value of the paper. Following your suggestion, we have carefully read the relevant studies by Ghaffar et al. (2025) and Zulkiffal et al. (2025), and have formally added them to the reference list of the revised manuscript as [5] and [6] (**see page 14**). These studies on AI-driven plant stress management and data-driven wheat improvement for rust resistance not only enrich our citation context but also provide a valuable complement to the potential applications of our pest identification technology within the broader context of agricultural intelligence.

11. Minor grammatical inconsistencies are present (e.g., “organically combines CNN and Transformer”). A careful language revision is recommended for clarity and fluency.

**Author response:** Thank you for your meticulous guidance regarding the language quality. Upon receiving your comments, we have thoroughly restructured and refined the grammar, logic, and phrasing of the entire manuscript, ensuring a fluent narrative and correcting informal expressions such as "organically combines." Since this revision involved a wide range of sentence structure adjustments, in order to maintain a clean overall layout, we have not used yellow highlighting to mark these changes. We sincerely apologize for any inconvenience this may cause during your review and kindly ask for your understanding.

12. The conclusion largely restates earlier content without offering forward-looking insights or practical implications. It could be strengthened by highlighting future research directions.

**Author response:** Thank you very much for this forward-looking suggestion. To enhance the value of the conclusion section, we have rewritten the "Conclusion" section in this revision (**see page 13**). The new version not only concisely summarizes the core contributions but also adds future research directions, making the conclusion more aligned with the positioning of a research paper with broad application prospects.

13. Although no human/animal subjects are involved, a brief clarification regarding data sourcing ethics (web-scraped images) would improve transparency.

**Author response:** Thank you for your rigorous requirements regarding academic ethics and transparency. Although our study does not involve human or animal subjects, to make the data sources more transparent, we have specifically added the "Ethics Statement" section in the revised manuscript (**see page 14**). In this statement, we elaborate in detail on the data collection methods, source attributions of the IP102 and D0 datasets, and the data usage terms we have complied with. Through this measure, we ensure that any ethical issues regarding web-scraped images are clearly explained and lawfully addressed.

14. The manuscript claims reduced computational burden but does not provide FLOPs, parameter count, or inference time comparisons, which are important for practical deployment.

**Author response:** Thank you for accurately pointing out the key metrics that must be evaluated for practical deployment of a model. Following your suggestion, we have added a detailed comparison table (**Table 3**) at the end of the "Performance comparison with state-of-the-art methods" subsection (**see pages 11 and 12**). This table specifically showcases the number of parameters, FLOPs, and inference time for our model compared to the baselines, accompanied by a corresponding analysis of the experimental data.

15. Some references are preprints (e.g., arXiv) and may need to be supplemented with peer-reviewed sources, as PLOS ONE encourages robust and verifiable citations.

**Author response:** Thank you very much for your attention to the rigor and verifiability of the references. We fully agree with PLOS ONE's high standards regarding cited sources. Upon receiving your comments, we conducted a detailed review of all cited literature. For the references [7], [17], [32], [33], and [36], which were originally labeled as preprints, we queried the latest academic databases. Except for references [32] and [33], which could not be updated as they are currently under review, we have successfully updated and replaced references [7], [17], and [36] with peer-reviewed versions published in relevant journals. We greatly appreciate you pushing us to improve the quality of our citations.

We deeply appreciate your time and insightful comments, which have significantly improved the quality of our manuscript. We hope the above revisions and responses meet your expectations.
