# Supplementary material for: CT-PestNet: A novel crop pest classification model based on cross-scale feature distillation enhancement and vision transformer with local token interaction
Source: PLoS One. 2026 Aug 14;21(8):e0356061. doi: 10.1371/journal.pone.0356061 (PMC13475986; doi:10.1371/journal.pone.0356061)
Supplement: Peer review history

# Peer review: Decision Letter 0

**DOI:** 10.1371/journal.pone.0356061.r001

**Authors:** Peng Geng

18 May 2026

PONE-D-26-13218

CT-PestNet: A Novel Crop Pest Classification Model Based on Cross-scale Feature Distillation Enhancement and Vision Transformer with Local Token Interaction

PLOS One

Dear Dr. Li,

Thank you for submitting your manuscript to PLOS ONE. After careful consideration, we feel that it has merit but does not fully meet PLOS ONE’s publication criteria as it currently stands. Therefore, we invite you to submit a revised version of the manuscript that addresses the points raised during the review process.

Please submit your revised manuscript by Jul 02 2026 11:59PM. If you will need more time than this to complete your revisions, please reply to this message or contact the journal office at plosone@plos.org. When you're ready to submit your revision, log on to https://www.editorialmanager.com/pone/ and select the 'Submissions Needing Revision' folder to locate your manuscript file.

Please include the following items when submitting your revised manuscript:

- A letter that responds to each point raised by the academic editor and reviewer(s). You should upload this letter as a separate file labeled 'Response to Reviewers'.
- A marked-up copy of your manuscript that highlights changes made to the original version. You should upload this as a separate file labeled 'Revised Manuscript with Track Changes'.
- An unmarked version of your revised paper without tracked changes. You should upload this as a separate file labeled 'Manuscript'.

If you would like to make changes to your financial disclosure, please include your updated statement in your cover letter. Guidelines for resubmitting your figure files are available below the reviewer comments at the end of this letter.

If applicable, we recommend that you deposit your laboratory protocols in protocols.io to enhance the reproducibility of your results. Protocols.io assigns your protocol its own identifier (DOI) so that it can be cited independently in the future. For instructions see: https://journals.plos.org/plosone/s/submission-guidelines#loc-laboratory-protocols. Additionally, PLOS ONE offers an option for publishing peer-reviewed Lab Protocol articles, which describe protocols hosted on protocols.io. Read more information on sharing protocols at https://plos.org/protocols?utm_medium=editorial-email&utm_source=authorletters&utm_campaign=protocols.

As the corresponding author, your ORCID iD is verified in the submission system and will appear in the published article. PLOS supports the use of ORCID, and we encourage all coauthors to register for an ORCID iD and use it as well. Please encourage your coauthors to verify their ORCID iD within the submission system before final acceptance, as unverified ORCID iDs will not appear in the published article. *Only*  the individual author can complete the verification step; PLOS staff *cannot*  verify ORCID iDs on behalf of authors.

We look forward to receiving your revised manuscript.

Kind regards,

Peng Geng

Academic Editor

PLOS One

Journal requirements:

When submitting your revision, we need you to address these additional requirements.

1. Please ensure that your manuscript meets PLOS ONE's style requirements, including those for file naming. The PLOS ONE style templates can be found at

https://journals.plos.org/plosone/s/file?id=wjVg/PLOSOne_formatting_sample_main_body.pdf and

https://journals.plos.org/plosone/s/file?id=ba62/PLOSOne_formatting_sample_title_authors_affiliations.pdf

2. Please note that PLOS One has specific guidelines on code sharing for submissions in which author-generated code underpins the findings in the manuscript. In these cases, we expect all author-generated code to be made available without restrictions upon publication of the work. Please review our guidelines at https://journals.plos.org/plosone/s/materials-and-software-sharing#loc-sharing-code and ensure that your code is shared in a way that follows best practice and facilitates reproducibility and reuse.

3. We note that Figures 1, 2, 4, 5, 9 and 10 in your submission contain copyrighted images. All PLOS content is published under the Creative Commons Attribution License (CC BY 4.0), which means that the manuscript, images, and Supporting Information files will be freely available online, and any third party is permitted to access, download, copy, distribute, and use these materials in any way, even commercially, with proper attribution. For more information, see our copyright guidelines: http://journals.plos.org/plosone/s/licenses-and-copyright.

We require you to either (1) present written permission from the copyright holder to publish these figures specifically under the CC BY 4.0 license, or (2) remove the figures from your submission:

1. You may seek permission from the original copyright holder of Figures 1, 2, 4, 5, 9 and 10 to publish the content specifically under the CC BY 4.0 license.

We recommend that you contact the original copyright holder with the Content Permission Form (http://journals.plos.org/plosone/s/file?id=7c09/content-permission-form.pdf) and the following text:

“I request permission for the open-access journal PLOS ONE to publish XXX under the Creative Commons Attribution License (CCAL) CC BY 4.0 (http://creativecommons.org/licenses/by/4.0/). Please be aware that this license allows unrestricted use and distribution, even commercially, by third parties. Please reply and provide explicit written permission to publish XXX under a CC BY license and complete the attached form.”

Please upload the completed Content Permission Form or other proof of granted permissions as an "Other" file with your submission.

In the figure caption of the copyrighted figure, please include the following text: “Reprinted from [ref] under a CC BY license, with permission from [name of publisher], original copyright [original copyright year].”

2. If you are unable to obtain permission from the original copyright holder to publish these figures under the CC BY 4.0 license or if the copyright holder’s requirements are incompatible with the CC BY 4.0 license, please either i) remove the figure or ii) supply a replacement figure that complies with the CC BY 4.0 license. Please check copyright information on all replacement figures and update the figure caption with source information. If applicable, please specify in the figure caption text when a figure is similar but not identical to the original image and is therefore for illustrative purposes only.

If the reviewer comments include a recommendation to cite specific previously published works, please review and evaluate these publications to determine whether they are relevant and should be cited. There is no requirement to cite these works unless the editor has indicated otherwise.

[Note: HTML markup is below. Please do not edit.]

Reviewers' comments:

Reviewer's Responses to Questions

**Comments to the Author**

1. Is the manuscript technically sound, and do the data support the conclusions?

The manuscript must describe a technically sound piece of scientific research with data that supports the conclusions. Experiments must have been conducted rigorously, with appropriate controls, replication, and sample sizes. The conclusions must be drawn appropriately based on the data presented.

Reviewer #1: Partly

Reviewer #2: Yes

**********

2. Has the statistical analysis been performed appropriately and rigorously?

Reviewer #1: No

Reviewer #2: I Don't Know

**********

3. Have the authors made all data underlying the findings in their manuscript fully available?

The PLOS Data policy requires authors to make all data underlying the findings described in their manuscript fully available without restriction, with rare exception (please refer to the Data Availability Statement in the manuscript PDF file). The data should be provided as part of the manuscript or its supporting information, or deposited to a public repository. For example, in addition to summary statistics, the data points behind means, medians and variance measures should be available. If there are restrictions on publicly sharing data—e.g. participant privacy or use of data from a third party—those must be specified.

Reviewer #1: No

Reviewer #2: Yes

**********

4. Is the manuscript presented in an intelligible fashion and written in standard English?

PLOS ONE does not copyedit accepted manuscripts, so the language in submitted articles must be clear, correct, and unambiguous. Any typographical or grammatical errors should be corrected at revision, so please note any specific errors here.

Reviewer #1: No

Reviewer #2: No

**********

5. Review Comments to the Author

Please use the space provided to explain your answers to the questions above. You may also include additional comments for the author, including concerns about dual publication, research ethics, or publication ethics. (Please upload your review as an attachment if it exceeds 20,000 characters)

Reviewer #1: Summary:

This paper presents a novel hybrid CNN-Transformer model for crop pest classification, with two interesting modules (CFDE for multi-scale feature distillation and LTIR for local token interaction). The experimental work is extensive and the results show clear improvements over several baselines. However, the manuscript has three major weaknesses that must be addressed before publication:

1. Lack of statistical analysis – no significance tests, no variance reporting.

2. Missing computational cost comparison – parameters, FLOPs, inference time.

3. Poor English – numerous grammatical errors and non-standard phrasing.

Specific recommendations for revision:

- Add a statistical comparison table with p‑values (e.g., McNemar’s test) for the main accuracy/F1 differences.

- Report results from at least 3 independent runs (mean ± std) for all models.

- Add a table comparing model size (parameters), FLOPs, and inference time per image.

- Provide the source code and a brief README for reproducibility.

- Undergo professional language editing.

- Add an ethics statement regarding the use of insect images (even if publicly sourced).

- Correct minor formatting issues (e.g., “Fig 1.(a)” → “Fig. 1(a)”, remove stray numbers on pages 17 and 9).

Overall recommendation: Minor revision – the scientific contribution is valid, but the presentation and statistical rigour need improvement.

Reviewer #2: The manuscript PONE-D-26-13218, titled “CT-PestNet: A Novel Crop Pest Classification Model Based on Cross-scale Feature Distillation Enhancement and Vision Transformer with Local Token Interaction” presents a well-structured and technically sound deep learning framework that integrates CNN and Transformer architectures for crop pest classification. The proposed CT-PestNet model, along with CFDE and LTIR modules, reflects a thoughtful attempt to address both global and local feature extraction challenges. The experimental validation on benchmark datasets (IP102 and D0) is comprehensive, and the inclusion of visualization analyses (e.g., Grad-CAM) strengthens the interpretability of the results. Overall, the study demonstrates promising performance improvements and contributes to the growing literature on AI-driven agricultural monitoring. However, the manuscript requires revision before it can be considered for publication. The following comments are provided to improve clarity, rigor, and alignment with PLOS ONE standards:

1. The abstract is informative but could be improved by including quantitative performance metrics (e.g., accuracy gains) to better highlight the contribution. Currently, claims such as “outperforms most existing models” remain too general.

2. Although limitations of CNN and Transformer models are mentioned, the precise research gap is not explicitly defined. It would be beneficial if the novelty of CFDE and LTIR is positioned more clearly against recent hybrid models.

3. The related work section primarily summarizes prior studies without critically analyzing their limitations. A comparative synthesis would strengthen the motivation for the proposed approach.

4. Design decisions such as kernel sizes (7×7, 5×5, 3×3) and token dimensions (D = 1024) are presented without justification. A short rationale or sensitivity analysis is recommended.

5. The manuscript reports accuracy and F1-score but lacks statistical significance testing or confidence intervals. It is suggested that variability across runs be reported.

6. Given the high performance on the D0 dataset (99.73% accuracy) , concerns regarding overfitting should be discussed, particularly considering dataset size.

7. The manuscript identifies class imbalance (e.g., fewer egg/larva samples) , yet no mitigation strategy (e.g., reweighting, augmentation) is implemented. This should be addressed experimentally.

8. While CFDE and LTIR contributions are evaluated, the study lacks component-level analysis (e.g., SENet contribution, kernel variation impact).

9. Some figures (e.g., Fig 6–7) are referenced without detailed interpretation in the text. Figure captions should be more self-contained and explanatory. Picture of rice stem fly in Figure 4 seems licensed as logo shows on it, please use your own pictures of these pests otherwise get permission from the original sources. Same comment must be dealt with for all other such pictures.

10. It is suggested that the authors cite recent relevant work to strengthen the manuscript’s broader context. In particular, Ghaffar et al. (2025) (https://doi.org/10.55627/pbiotech.003.04.1702) on AI-driven plant stress management and Zulkiffal et al. (2025) (https://doi.org/10.55627/pbiotech.003.04.1709) on data-driven wheat improvement for rust resistance provide useful complementary perspectives. Their inclusion would enhance the interdisciplinary relevance of the study.

11. Minor grammatical inconsistencies are present (e.g., “organically combines CNN and Transformer” ). A careful language revision is recommended for clarity and fluency.

12. The conclusion largely restates earlier content without offering forward-looking insights or practical implications. It could be strengthened by highlighting future research directions.

13. Although no human/animal subjects are involved, a brief clarification regarding data sourcing ethics (web-scraped images) would improve transparency.

14. The manuscript claims reduced computational burden but does not provide FLOPs, parameter count, or inference time comparisons, which are important for practical deployment.

15. Some references are preprints (e.g., arXiv) and may need to be supplemented with peer-reviewed sources, as PLOS ONE encourages robust and verifiable citations.

**********

6. PLOS authors have the option to publish the peer review history of their article (what does this mean?). If published, this will include your full peer review and any attached files.

If you choose “no”, your identity will remain anonymous but your review may still be made public.

**Do you want your identity to be public for this peer review?**  For information about this choice, including consent withdrawal, please see our Privacy Policy.

Reviewer #1: No

Reviewer #2: No

**********

[NOTE: If reviewer comments were submitted as an attachment file, they will be attached to this email and accessible via the submission site. Please log into your account, locate the manuscript record, and check for the action link "View Attachments". If this link does not appear, there are no attachment files.]

To ensure your figures meet our technical requirements, please review our figure guidelines: https://journals.plos.org/plosone/s/figures

You may also use PLOS’s free figure tool, NAAS, to help you prepare publication quality figures: https://journals.plos.org/plosone/s/figures#loc-tools-for-figure-preparation.

NAAS will assess whether your figures meet our technical requirements by comparing each figure against our figure specifications.

# Author comment: Author response to Decision Letter 1

**DOI:** 10.1371/journal.pone.0356061.r002

30 Jun 2026

We deeply appreciate your time and insightful comments, which have significantly improved the quality of our manuscript. We hope the above revisions and responses meet your expectations. We have submitted the revised manuscript and a document detailing our point-by-point response to the journal requirements and reviewers' comments. We kindly ask the reviewers and the editorial team to review the revisions and hope for your approval.

Attachment Submitted filename: Response to Reviewers.docx

# Peer review: Decision Letter 1

**DOI:** 10.1371/journal.pone.0356061.r003

**Authors:** Peng Geng

30 Jul 2026

CT-PestNet: A Novel Crop Pest Classification Model Based on Cross-scale Feature Distillation Enhancement and Vision Transformer with Local Token Interaction

PONE-D-26-13218R1

Dear Dr. Li,

We’re pleased to inform you that your manuscript has been judged scientifically suitable for publication and will be formally accepted for publication once it meets all outstanding technical requirements.

Within one week, you’ll receive an e-mail detailing the required amendments. When these have been addressed, you’ll receive a formal acceptance letter and your manuscript will be scheduled for publication.

An invoice will be generated when your article is formally accepted. Please note, if your institution has a publishing partnership with PLOS and your article meets the relevant criteria, all or part of your publication costs will be covered. Please make sure your user information is up-to-date by logging into Editorial Manager at Editorial Manager® and clicking the ‘Update My Information' link at the top of the page. For questions related to billing, please contact billing support.

If your institution or institutions have a press office, please notify them about your upcoming paper to help maximize its impact. If they’ll be preparing press materials, please inform our press team as soon as possible -- no later than 48 hours after receiving the formal acceptance. Your manuscript will remain under strict press embargo until 2 pm Eastern Time on the date of publication. For more information, please contact onepress@plos.org.

Kind regards,

Peng Geng

Academic Editor

PLOS One

Additional Editor Comments (optional):

Reviewers' comments:

Reviewer's Responses to Questions

**Comments to the Author**

1. If the authors have adequately addressed your comments raised in a previous round of review and you feel that this manuscript is now acceptable for publication, you may indicate that here to bypass the “Comments to the Author” section, enter your conflict of interest statement in the “Confidential to Editor” section, and submit your "Accept" recommendation.

Reviewer #1: (No Response)

Reviewer #2: All comments have been addressed

**********

2. Is the manuscript technically sound, and do the data support the conclusions?

The manuscript must describe a technically sound piece of scientific research with data that supports the conclusions. Experiments must have been conducted rigorously, with appropriate controls, replication, and sample sizes. The conclusions must be drawn appropriately based on the data presented.

Reviewer #1: (No Response)

Reviewer #2: Yes

**********

3. Has the statistical analysis been performed appropriately and rigorously?

Reviewer #1: (No Response)

Reviewer #2: Yes

**********

4. Have the authors made all data underlying the findings in their manuscript fully available?

The PLOS Data policy requires authors to make all data underlying the findings described in their manuscript fully available without restriction, with rare exception (please refer to the Data Availability Statement in the manuscript PDF file). The data should be provided as part of the manuscript or its supporting information, or deposited to a public repository. For example, in addition to summary statistics, the data points behind means, medians and variance measures should be available. If there are restrictions on publicly sharing data—e.g. participant privacy or use of data from a third party—those must be specified.

Reviewer #1: Yes

Reviewer #2: Yes

**********

5. Is the manuscript presented in an intelligible fashion and written in standard English?

PLOS ONE does not copyedit accepted manuscripts, so the language in submitted articles must be clear, correct, and unambiguous. Any typographical or grammatical errors should be corrected at revision, so please note any specific errors here.

Reviewer #1: Yes

Reviewer #2: Yes

**********

6. Review Comments to the Author

Please use the space provided to explain your answers to the questions above. You may also include additional comments for the author, including concerns about dual publication, research ethics, or publication ethics. (Please upload your review as an attachment if it exceeds 20,000 characters)

Reviewer #1: The authors have comprehensively addressed all concerns raised in my previous review:

1. Technical completeness – Provided detailed mathematical formulations and architectural diagrams for CFDE and LTIR.

2. Experimental rigor – Added statistical significance testing (paired t-tests, p-values), standard deviations from five independent runs, and error bars in figures.

3. Computational analysis – Added Table 3 comparing parameters, FLOPs, and inference time.

4. Data availability – Corrected all data URLs and added an Ethics Statement clarifying data sources and usage terms.

5. Code sharing – Explained institutional confidentiality restrictions and offered private access for reviewers.

6. Language quality – Thoroughly revised and edited the manuscript for clarity and fluency.

7. Ablation studies – Added detailed component-level analysis including SENet contribution and kernel size variations.

8. Limitations – Acknowledged class imbalance challenges and proposed future research directions.

Final Recommendation

I recommend ACCEPTANCE of the manuscript in its current form.

The revised manuscript is technically sound, methodologically rigorous, ethically transparent, and well-written. It makes a meaningful contribution to the field of AI-driven crop pest classification and meets all PLOS ONE publication standards.

Reviewer #2: The authors have significantly improved improved the manuscript, I endorse its its publication in PLoS One.

**********

7. PLOS authors have the option to publish the peer review history of their article (what does this mean?). If published, this will include your full peer review and any attached files.

If you choose “no”, your identity will remain anonymous but your review may still be made public.

**Do you want your identity to be public for this peer review?**  For information about this choice, including consent withdrawal, please see our Privacy Policy.

Reviewer #1: **Yes:** Dr. Nabil Abo Kaf

Reviewer #2: No

**********

# Editor report: Acceptance letter

**DOI:** 10.1371/journal.pone.0356061.r004

**Authors:** Peng Geng

PONE-D-26-13218R1

PLOS One

Dear Dr. Li,

I'm pleased to inform you that your manuscript has been deemed suitable for publication in PLOS One. Congratulations! Your manuscript is now being handed over to our production team.

At this stage, our production department will prepare your paper for publication. This includes ensuring the following:

* All references, tables, and figures are properly cited

* All relevant supporting information is included in the manuscript submission,

* There are no issues that prevent the paper from being properly typeset

You will receive further instructions from the production team, including instructions on how to review your proof when it is ready. Please keep in mind that we are working through a large volume of accepted articles, so please give us a few days to review your paper and let you know the next and final steps.

Lastly, if your institution or institutions have a press office, please let them know about your upcoming paper now to help maximize its impact. If they'll be preparing press materials, please inform our press team within the next 48 hours. Your manuscript will remain under strict press embargo until 2 pm Eastern Time on the date of publication. For more information, please contact onepress@plos.org.

You will receive an invoice from PLOS for your publication fee after your manuscript has reached the completed accept phase. If you receive an email requesting payment before acceptance or for any other service, this may be a phishing scheme. Learn how to identify phishing emails and protect your accounts at https://explore.plos.org/phishing.

If we can help with anything else, please email us at customercare@plos.org.

Thank you for submitting your work to PLOS ONE and supporting open access.

Kind regards,

PLOS ONE Editorial Office Staff

on behalf of

Dr. Peng Geng

Academic Editor

PLOS One
